# Real-World Comparisons of Low-Dose NOACs versus Standard-Dose NOACs or Warfarin on Efficacy and Safety in Patients with AF: A Meta-Analysis

**DOI:** 10.1155/2022/4713826

**Published:** 2022-03-07

**Authors:** Ze Li, Xiaozhen Wang, Dandan Li, Aiping Wen

**Affiliations:** ^1^Department of Pharmacy, Beijing Friendship Hospital, Capital Medical University, Beijing, China; ^2^Central Laboratory, Xuanwu Hospital, Capital Medical University, Beijing, China

## Abstract

**Objective:**

We aimed to further investigate the efficacy and safety of low-dose NOACs by performing a meta-analysis of cohort studies.

**Background:**

Meta-analyses of randomized controlled trials (RCTs) have demonstrated that low-dose non-vitamin K antagonist oral anticoagulants (NOACs) showed inferior efficacy compared with standard-dose NOACs, although they are still frequently prescribed for patients with atrial fibrillation (AF) in the clinical practice.

**Methods:**

Cochrane Central Register of Controlled Trials (CENTRAL), Embase, and MEDLINE were systematically searched from the inception to September 9, 2021, for cohort studies that compared the efficacy and/or safety of low-dose NOACs in patients with AF. The primary outcomes were ischemic stroke and major bleeding, and the secondary outcomes were mortality, intracranial hemorrhage (ICH), and gastrointestinal hemorrhage (GH). Hazard ratios (HRs) and 95% confidence intervals (CIs) were estimated with the random-effect model.

**Results:**

Twenty-five publications involving 487856 patients with AF were included. Compared with standard-dose NOACs, low-dose NOACs had comparable risks of ischemic stroke (HR = 1.03, 95% CI 0.96 to 1.11), major bleeding (HR = 1.12, 95% CI 0.97 to 1.28), ICH (HR = 1.09, 95% CI 0.88 to 1.36), and GH (HR = 1.11, 95% CI 0.92 to 1.33), except for a higher risk of mortality (HR = 1.41, 95% CI 1.21 to 1.65). Compared with warfarin, low-dose NOACs were associated with lower risks of ischemic stroke (HR = 0.72, 95% CI .67 to 0.78), mortality (HR = 0.67, 95% CI 0.59 to 0.77), major bleeding (HR = 0.64, 95% CI 0.53 to 0.79), ICH (HR = 0.57, 95% CI 0.42 to 0.77), and GH (HR = 0.78, 95% CI 0.64 to 0.95).

**Conclusions:**

Low-dose NOACs were comparable to standard-dose NOACs considering risks of ischemic stroke, major bleeding, ICH, and GH, and they were superior to warfarin. Low-dose NOACs might be prescribed effectively and safely for patients with AF. Considering limitations, further well-designed prospective studies are foreseen.

## 1. Introduction

Atrial fibrillation (AF), known as a common cardiac arrhythmia worldwide, can cause ischemic stroke and seriously jeopardize the health of global elder patients [1]. For decades, warfarin was prescribed to prevent ischemic stroke from AF by decreasing the production of several clotting proteins that rely on vitamin K [2]. However, the adherence to warfarin is severely affected by the frequent international normalized ratio (INR) monitoring, drug-drug and drug-food interactions [3]. In recent years, the approval of non-vitamin K antagonist oral anticoagulants (NOACs), which directly inhibit the critical factors of the coagulation cascade, provided new anticoagulant strategies for the patients with AF.

Meta-analyses of RCTs assessed the efficacy and safety of standard-dose NOACs, low-dose NOACs, and warfarin in patients with AF. Moreover, the results revealed that low-dose NOACs were inferior to standard-dose NOACs in the efficacy with a higher risk of ischemic stroke and had no superior efficacy and safety than warfarin [4, 5]; standard-dose NOACs were superior to warfarin in the efficacy and safety with less ischemic stroke, mortality, and ICH [4–6]. However, low-dose NOACs are still frequently prescribed for patients with AF. Low-dose NOACs were prescribed for 31%, 19%, and 29% of patients in Korea [7], France [8], and America [9], respectively. RCTs were performed under optimized conditions, strict inclusion and exclusion criteria, which might not reflect real-world conditions. Moreover, RCTs enroll a small, nonrepresentative subset of patients and overlook the important interactions between the patients and the real world, which can affect treatment outcomes [10]. Furthermore, medication adherence, the key point for treatment effectiveness, is closely monitored in RCTs, which is not always the case in clinical practice [10]. Real-world cohort studies, which enroll patients of broad-spectrum baseline characteristics, may provide a comprehensive picture of the clinical practice. Therefore, we aimed to further investigate the efficacy and safety of low-dose NOACs by conducting a meta-analysis of all relevant cohort studies.

## 2. Methods

This meta-analysis was prepared according to the PRISMA (Preferred Reporting Items for Systemic Reviews and Meta-analysis) and MOOSE (Meta-Analysis of Observational Studies in Epidemiology) guidelines [11, 12].

### 2.1. Search Strategy and Study Selection

Cochrane Central Register of Controlled Trials (CENTRAL) (from inception to September 9, 2021), MEDLINE (from inception to September 9, 2021), and Embase (from inception to September 9, 2021) were systematically searched. Details of the search strategy are illustrated in Supplementary Tables S1–S3.

We developed inclusion criteria for this meta-analysis prospectively. The criteria of studies screening were as follows: (1) the target population was patients with AF; (2) studies involved lose-dose NOACs and standard-dose NOACs or warfarin; (3) studies included efficacy (ischemic stroke and mortality) or safety outcomes (major bleeding, intracranial hemorrhage, and gastrointestinal hemorrhage); (4) the study type was the cohort. AF patients with valvular heart disease (VHD) or receiving NOACs after catheter ablation and studies published in the forms of conference abstracts, letters, or protocols were excluded. In addition, for the same data source or overlapping data reported in more than one study, only the most comprehensive data with the longest follow-up period was included. References of included studies and relevant meta-analyses were screened for additional eligible studies as well.

### 2.2. Definitions of Low-Dose NOACs, Standard-Dose NOACs, and Warfarin

Definitions were in accordance with the included studies. Standard-dose NOACs and warfarin were defined as dabigatran 150 mg b.i.d., rivaroxaban 20 mg q.d., apixaban 5 mg b.i.d., and edoxaban 60 mg q.d., and INR of 2.0–3.0 [13]. Low-dose NOACs were defined as dabigatran 110/75 mg b.i.d., rivaroxaban 15/10 mg q.d., apixaban 2.5 mg b.i.d., and edoxaban 30 mg q.d. [4, 9]. And for patients with creatinine clearance rate (CrCl) of 30–50 mL/min, age ≥70 years, and a prior history of bleeding, standard-dose dabigatran was defined as 110 mg b.i.d. [14, 15]; for patients with CrCl of 15–50 mL/min, standard-dose rivaroxaban was defined as 10 mg q.d. [16, 17]; for patients with any two of the following characteristics, ≥ 80 years old, bodyweight <60 kg, and serum creatinine level (Cr) ≥ 1.5 mg/dL, standard-dose apixaban was defined as 2.5 mg b.i.d. [18, 19]; for patients with CrCl of 15–50 mL/min or bodyweight <60 kg, standard-dose edoxaban was defined as 30 mg q.d. [20].

### 2.3. Data Extraction and Quality Assessment

The primary efficacy outcome was ischemic stroke, and the secondary efficacy outcome was mortality (all-cause mortality). The primary safety outcome was major bleeding, defined as fatal bleeding or bleeding in a critical site, and the secondary safety outcomes were intracranial hemorrhage (ICH) and gastrointestinal hemorrhage (GH).

Two reviewers (Ze Li and Xiaozhen Wang) independently screened titles and abstracts of the retrieved studies to exclude those that did not explore questions of interest and then independently screened full texts of the remaining studies to identify those that met all the inclusion criteria. We manually checked the reference list of each acquired article for relevant studies. For each included study, two reviewers independently extracted the characteristics of the included studies and patients, as well as outcomes as predefined. Discrepancies were resolved by discussing with the third reviewer (Aiping Wen).

Bias risks were assessed with the Newcastle-Ottawa quality assessment scale [21]. The publication bias was quantitatively assessed by the Begg's [22] and Egger's tests [23], *P* < 0.05 was taken as statistically significant. Two reviewers (Ze Li and Xiaozhen Wang) assessed risks of bias independently and in duplicate. Any disagreements were resolved in consultation with the supervisor (Aiping Wen).

### 2.4. Data Synthesis and Statistical Analysis

Intention to treat analysis (ITT) results were used wherever possible. If ITT results were not available, we used the data the author reported. All analyses were performed by Stata 16.0. Hazard ratios (HRs) and corresponding 95% confidence intervals (CIs) were estimated with the random-effect model. The heterogeneity among studies was assessed by *I*^*2*^ with <25%, 25–50%, and >50% indicating low, moderate, and high degree of heterogeneity, respectively. Meta-regression analyses were performed to examine possible sources of heterogeneity in the data.

To explore the influence for different regions of patients, subgroup meta-analyses were performed by stratifying patients into Asia and non-Asia. Most cohort studies used the propensity score matching (PSM) method or multivariable model (MM) to balance the confounding factors between groups and minimize the heterogeneity, so we enrolled adjusted cohort studies to perform adjusted subgroup meta-analyses. For all comparisons in this meta-analysis, *P* < 0.05 was taken as statistically significant.

## 3. Results

### 3.1. Studies Identification and Characteristics

**Figure 1:**
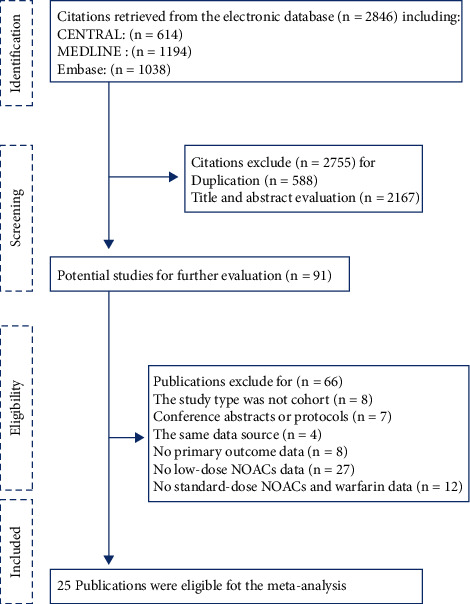
Flow-chart for the selection of included studies.

A total of 2846 publications were identified through database search (Figure 1). After the study screening process, twenty-five cohort studies meeting the inclusion criteria were included.

In general, there were 487856 patients in all enrolled studies. 238292 patients were included in the standard-dose group, including 115518 patients receiving NOACs and 122774 patients receiving warfarin, and 249564 patients were involved in the low-dose NOACs group. The baseline characteristics of included studies are shown in Table 1. The detailed previous medical history and group contents of included studies are illustrated in Supplementary Tables S4 and S5.

### 3.2. Risks of Bias Assessment

Results of bias assessments are summarized in Supplementary Tables S6 and S7. Overall, most cohort studies reported low risks of bias, while seven studies did not balance the confounding factors between groups, which had risks of comparability bias [8, 25, 30, 31, 39–41]. Three studies did not report the length of follow-up [27, 31, 35], and most studies did not show the lost follow-up rate, which had risks of outcome bias. In addition, there was no publication bias for this meta-analysis by the Begg's and Egger's tests, except for the risk of ICH (*P* = 0.035, Egger's test) in the comparison of low-dose NOACs versus warfarin.

### 3.3. Low-Dose NOACs versus Standard-Dose NOACs

**Figure 2:**
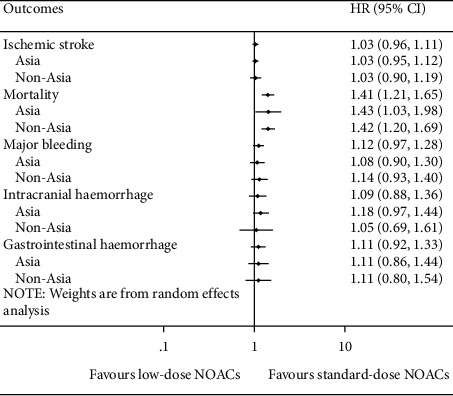
Meta-analyses of the efficacy and safety for low-dose NOACs versus standard-dose NOACs. HR = hazard ratio and CI = confidence interval.

There was no significant difference between low-dose NOACs and standard-dose NOACs for risks of ischemic stroke (HR = 1.03, 95% CI 0.96 to 1.11, *I*^*2*^ = 0%), major bleeding (HR = 1.12, 95% CI 0.97 to 1.28, *I*^*2*^ = 52.3%), ICH (HR = 1.09, 95% CI 0.88 to 1.36, *I*^*2*^ = 33.2%), and GH (HR = 1.11, 95% CI 0.92 to 1.33, *I*^*2*^ = 65.0%). However, compared with standard-dose NOACs, low-dose NOACs were associated with a higher risk of mortality (HR = 1.41, 95% CI 1.21 to 1.65, *I*^*2*^ = 78.2%). And results of Asia and non-Asia subgroup meta-analyses were also the same to the overall (Figure 2). Details of subgroup meta-analyses are illustrated in Supplementary Figures S1–S5.

To minimize the heterogeneity and obtain more reliable results, adjusted subgroup meta-analyses including cohort studies with the PSM or MM method were performed. Results of all outcomes were consistent with the overall meta-analysis as well. Details of adjusted subgroup meta-analyses are illustrated in Supplementary Figures S6–S9.

For meta-regression analyses, no significant correlations were observed in most efficacy and safety outcomes. However, a significant correlation was found between major bleeding and mean age (*P* = 0.010), with HR increasing as the mean age of included patients ascended (Supplementary Figure S10); other significant predictors of HR were found between ICH, mean age (*P* = 0.046), and female (*P* = 0.035) as well, with HR increasing as the mean age (Supplementary Figure S11) or female percent of included patients ascended (Supplementary Figure S12). Details of meta-regression analyses are illustrated in Supplementary Table S8.

To balance the confounding factors, subgroup meta-analyses stratified by mean age (divided into older and younger groups by median) were performed, respectively. In general, all results were consistent with the overall meta-analysis. Details of subgroup meta-analyses are shown in Supplementary Figures S13 and S14.

### 3.4. Low-Dose NOACs versus Warfarin

**Figure 3:**
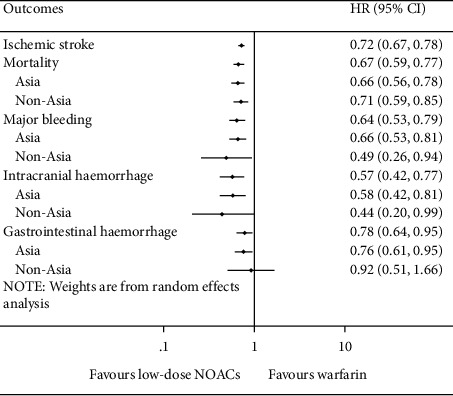
Meta-analyses of the efficacy and safety for low-dose NOACs versus warfarin. HR = hazard ratio and CI = confidence interval.

Compared with warfarin, low-dose NOACs were associated with lower risks of ischemic stroke (HR = 0.72, 95% CI .67 to 0.78, *I*^*2*^ = 2.1%), mortality (HR = 0.67, 95% CI 0.59 to 0.77, *I*^*2*^ = 77.8%), major bleeding (HR = 0.64, 95% CI 0.53 to 0.79, *I*^*2*^ = 71.8%), ICH (HR = 0.57, 95% CI 0.42 to 0.77, *I*^*2*^ = 69.5%), and GH (HR = 0.78, 95% CI 0.64 to 0.95, *I*^*2*^ = 45.6%). And results of Asia and non-Asia subgroup meta-analyses were similar to the overall except for the comparable risk of GH (HR = 0.92, 95% CI 0.51 to 1.66, *I*^*2*^ = 0%) in non-Asia (Figure 3). Details of subgroup meta-analyses are shown in Supplementary Figures S15–S19.

Results of adjusted subgroup meta-analyses were consistent with the overall as well. Details of adjusted subgroup meta-analyses are illustrated in Supplementary Figures S20 and S21. For meta-regression analyses, no significant correlations were observed in efficacy and safety outcomes. Details of meta-regression analyses are illustrated in Supplementary Table S9.

## 4. Discussion

To the best of our knowledge, this is the first meta-analysis of cohort studies for low-dose NOACs versus standard-dose NOACs or warfarin in patients with AF. A few previous meta-analyses had tried to assess the efficacy and safety of low-dose NOACs by RCTs [4, 5]. And the results indicated that when compared with standard-dose NOACs, low-dose NOACs showed the inferior efficacy with a higher risk of ischemic stroke; when compared with warfarin, low-dose NOACs showed the comparable efficacy and safety. Even though the meta-analysis of RCTs is the highest level of evidence, results of cohort studies may better represent the clinical practice with additional real-world data. For example, the previous meta-analyses of RCTs only enrolled patients of approximately 70 years old with the standard weight of roughly 66 kg [4–6]. These may not be generalizable to underrepresented patients, such as the patients with low weight, older age, or who were not yet represented in RCTs, so we performed this meta-analysis.

Our meta-analysis revealed that compared with standard-dose NOACs, low-dose NOACs had comparable risks of ischemic stroke and bleeding (including major bleeding, ICH, and GH), except for a higher risk of mortality; compared with warfarin, low-dose NOACs showed lower risks of ischemic stroke, mortality, and bleeding. To assess the influence of different regions, we stratified the patients into the Asia subgroup and non-Asia subgroup. Results of subgroup meta-analyses were consistent with the overall except for the comparable risk of GH for the non-Asia subgroup in the comparison of low-dose NOACs versus warfarin.

We need to note that the baseline characteristics of cohort studies may be diverse compared to RCTs. Concerning some included studies, the mean or median ages of low-dose NOACs group were much older than standard-dose NOACs group, which led to the relatively lower CrCL, higher CHA_2_DS_2_-VASc and HAS-BLED scores [8, 24, 26, 32, 40, 42–45]. Moreover, there were some heterogeneities in the previous medical history, including hypertension, diabetes, heart failure, vascular disease, stroke/transient ischemic attack (TIA), and major bleeding. Due to the broad-spectrum baseline characteristics, most cohort studies used the PSM or MM method to adjust the data and minimize the heterogeneity. Adjusted subgroup meta-analyses including cohort studies with PSM or MM were performed as well, and the results were consistent with the overall meta-analysis.

Meta-regression analyses indicated that the mean age and female percent of included patients captured a very substantial portion of the heterogeneity in the data, so subgroup meta-analyses stratified by those were performed to balance the confounding factors. Similarly, the results were consistent with the overall as well. Nonetheless, considering the relatively few studies and ineluctable heterogeneity in this meta-analysis, further high qualified prospective studies are required to validate these results.

Most of our results were similar to the previous meta-analyses of RCTs. However, there were some conflicting results in our meta-analysis compared with RCTs, such as the comparable risk of ischemic stroke and higher risk of mortality in the comparison of standard-dose NOACs, and lower risks of ischemic stroke, major bleeding, and GH in the comparison of warfarin [4, 5]. The difference in outcomes could be partially explained by several reasons: firstly, the patients' baselines of RCTs were narrow and nonrepresentative, with the approximate age of 70 years old, bodyweight of 66 kg, female percent of 40%, and CHADS_2_ score of 2.0–3.0 [4, 5]. These might only address a special population of AF patients. On the contrary, cohort studies in this meta-analysis presented broad-spectrum baseline characteristics, with age ranging from 63.3 to 88.7 years old, BMI ranging from 23.1 to 31.7, CHA_2_DS_2_-VASc and HAS-BLED scores ranging from 1.9 to 5.2, 0.8 to 3.0, respectively. Compared with RCTs, cohort studies involved the individual of older age, lower body weight and CrCL, or higher CHA_2_DS_2_-VASc and HAS-BLED scores, who might be more susceptible to low-dose NOACs. Secondly, the adherence to standard-dose NOAC was about 60%, and more than one-third of patients with label NOAC prescription received a reduced low-dose [46]. As a result, some patients might be prescribed for standard-dose NOACs, whereas they take low-dose NOACs in reality. We believed that the above two reasons might contribute to the noninferiority of low-dose NOACs versus standard-dose NOACs in the real world. Thirdly, the mean or median ages of low-dose NOACs were much older than standard-dose NOACs in nine studies [8, 24, 26, 32, 40, 42–45], and we considered this might explain the higher risk of mortality for low-dose NOACs. As another study showed, the older patients with AF were faced more comorbidities and death factors [47], which might eventually result in the higher risk of mortality. In addition, as it was not convenient to monitor the quality of warfarin routine usage, and many patients cannot reach the baseline requirement of time in therapeutic range (TTR) [48]; this might lead to the superiority of low-dose NOACs versus warfarin in clinical practice.

Warfarin showed some therapeutic limitations in the clinical practice, whose effect was widely affected by food and drugs, and patients need to monitor the INR frequently to supervise the efficacy and risk of major bleeding [49]. Major bleeding can seriously affect the anticoagulation treatment, such as higher risks of stroke and mortality [50], longer hospitalization [51], and more health care resource utilization [52]. At the same time, patients taking warfarin often had less time within the therapeutic range [48]. In this meta-analysis, low-dose NOACs were noninferior to standard-dose NOACs and superior to warfarin. Thus, considering the excellence and convenience, low-dose NOACs could be an effective and safe alternative to warfarin.

### 4.1. Limitations

However, there were some potential limitations for our meta-analysis. Firstly, due to the limited number of included studies, we pooled all NOACs together even though rivaroxaban, apixaban, and edoxaban are the factor Xa inhibitors [53], while dabigatran is the thrombin inhibitor [54], which was conducted in another meta-analysis [55]. This may not cause significant bias, for they are all direct-acting oral anticoagulants inhibiting the critical factors in the coagulation cascade. Secondly, this meta-analysis might have some fundamental heterogeneity due to the nature of cohort studies, such as the mean age, CHA_2_DS_2_-VASc and HAS-BLED scores. However, most studies had used the PSM or MM method to adjust the data and minimize the heterogeneity. In addition, the results of adjusted subgroup meta-analyses including studies with PSM or MM were consistent with the overall as well. Thirdly, most included studies did not report the quality of TTR for warfarin. As the efficacy of warfarin was affected by the TTR, many patients cannot reach the baseline requirement of TTR [48], which might lead to the unexpected bias of low-dose NOACs versus warfarin. This limitation could be found in other meta-analyses involving warfarin [55, 56]. However, the effectiveness of the treatment is ensured not only by effective and potent drugs, but also by patients' adherence to the therapy [57], and we should have various and comprehensive views of this limitation.

## 5. Conclusions

In general, for patients with AF, this meta-analysis of cohort studies demonstrated that low-dose NOACs were comparable to standard-dose NOACs considering risks of ischemic stroke, major bleeding, ICH, and GH, and they were superior to warfarin. Thus, low-dose NOACs might be prescribed effectively and safely for patients with AF. However, considering limitations, further well-designed prospective studies are required to validate these results.

## Figures and Tables

**Table 1:** Patient baseline characteristics of included studies.

Author, year	Region	Study type	Adjusted method	Group	Sample size	Age (years)	Female (%)	Follow-up (Months)	BMI (kg/m^2^)	CHA_2_DS_2_-VASc	HAS-BLED	CrCl (mL/min)
Murata N 2019 [24]	Japan	Cohort	PSM	Standard-dose	746	66.9 ± 9.0	21.6	43.6	25.0 ± 4.0	2.42 ± 1.39	1.16 ± 0.85	84.1 ± 27.5
				Low-dose	369	71.2 ± 8.2	29.0		24.5 ± 3.8	2.88 ± 1.39	1.25 ± 0.78	70.1 ± 21.2
Wakamatsu Y 2020 [25]	Japan	Cohort	NR	Standard-dose	749	63.3 ± 9.4	23.0	25.7	24.7 ± 3.7	2.10 ± 1.50	0.80 ± 0.80	76.7 ± 23.8
				Low-dose	216	64.8 ± 9.5	34.3		24.2 ± 3.4	2.40 ± 1.60	0.90 ± 0.80	73.3 ± 22.3
Ohno J 2021 [26]	Japan	Cohort	PSM	Standard-dose	907	66.0 ± 10.0	23.3	26.5	25.0 ± 4.0	2.74	2.27	82.8
				Low-dose	338	70.0 ± 10.0	34.9		24.0 ± 4.0	3.23	2.54	73.5
Lee HF 2018 [27]	Taiwan	Cohort	PSM	Low-dose	26000	78.0 ± 10.0	48.0	NR	NR	4.02 ± 1.29	2.98 ± 0.92	NR
				Warfarin	16000	78.0 ± 10.0	48.0			4.01 ± 1.28	2.99 ± 0.90	
Yu HT 2018 [28]	Korea	Cohort	PSM	Low-dose	3016	72.8 ± 9.1	48.0	5.0^c^	NR	4.90 ± 1.80	NR	NR
				Warfarin	3016	72.6 ± 9.9	46.7			4.80 ± 2.00		
Chan YH 2018 [29]	Taiwan	Cohort	PSM	Standard-dose^a^	6307	76.0 ± 10.0	45.0	35.2	NR	3.89 ± 0.84	2.96 ± 0.61	NR
				Low-dose^a^	47392							
				Warfarin	19375	76.0 ± 10.0	46.0			3.89 ± 0.88	2.97 ± 0.61	
Chang HK 2016 [30]	Korea	Cohort	NR	Standard-dose^a^	51	84.2 ± 3.5	60.1	24.9	24.4 ± 3.6	4.70 ± 1.40	2.60 ± 1.00	51.0 ± 13.9
				Low-dose^a^	97							
				Warfarin	145	83.2 ± 3.1	59.3		23.7 ± 3.6	4.70 ± 1.40	2.40 ± 0.90	53.1 ± 17.4
Akagi Y 2019 [31]	Japan	Cohort	NR	Standard-dose^a^	187	70.8 ± 10.8	34.2	NR	NR	1.92 ± 1.33^b^	NR	69.4 ± 25.3
				Low-dose^a^	488							
Yu HT 2020 [7]	Korea	Cohort	PSM	Standard-dose	32400	69.8 ± 9.5	38.2	36.0	NR	4.60 ± 1.70	NR	NR
				Low-dose	16757	70.7 ± 7.9	39.0			4.50 ± 1.80		
Cho MS 2019 [32]	Korea	Cohort	PSM	Low-dose	29695	73.8 ± 8.8	49.1	15.0	24.6 ± 2.9	3.60 ± 1.20	2.50 ± 0.90	NR
				Warfarin	10409	70.8 ± 11.0	46.0		24.4 ± 2.8	3.50 ± 1.20	2.60 ± 1.00	
Jeong HK 2019 [33]	Korea	Cohort	PSM	Low-dose	414	71.4 ± 10.5	36.7	12.0	NR	3.30 ± 1.80	NR	85.4
				Warfarin	804	70.4 ± 10.2	39.6			3.40 ± 1.80		87.0
Kohsaka S 2020 [34]	Japan	Cohort	PSM	Low-dose	17481	76.2 ± 10.6	38.9	28.9	NR^d^	3.80 ± 1.90	NR	NR
				Warfarin	19059	76.1 ± 11.9	38.8			3.80 ± 2.10		
Kohsaka S 2017 [35]	Japan	Cohort	PSM	Low-dose	6726	75.8 ± 10.0	38.9	NR	23.3 ± 4.5	3.30 ± 1.60	NR	NR
				Warfarin	6726	76.2 ± 10.5	38.0		23.1 ± 4.2	3.40 ± 1.60		
Lai CL 2018 [36]	Taiwan	Cohort	PSM	Low-dose	1489	88.4 ± 2.9	48.6	6.6	NR	3.80 ± 1.30	NR	NR
				Warfarin	1497	88.7 ± 3.1	54.8			3.80 ± 1.20		
Lee SR 2019 [37]	Korea	Cohort	PSM	Standard-dose	5196	71.2 ± 8.1	45.1	30.0	24.7 ± 3.3	3.50 ± 1.60	NR	82.5 ± 37.5
				Low-dose	5777	72.1 ± 8.4	44.9		24.5 ± 3.5	3.60 ± 1.60		81.5 ± 49.6
				Warfarin	5777	72.2 ± 8.9	46.5		24.5 ± 3.4	3.70 ± 1.80		81.3 ± 41.3
Chan YH 2019 [38]	Taiwan	Cohort	PSM	Low-dose	60212	74.7 ± 10.7	42.6	16.0	NR	3.60 ± 0.70	2.60 ± 0.50	NR
				Warfarin	19761	74.6 ± 10.7	43.3			3.60 ± 0.80	2.70 ± 0.50	
Alcusky M 2018 [39]	America	Cohort	NR	Standard-dose	374	82.0^c^	66.6	3.9^c^	NR	NR^d^	NR	NR
				Low-dose	336	86.5^c^	78.0					
Bouget J 2020 [8]	France	Cohort	PSM	Standard-dose	17659	67.0^c^	43.3	7.8^c^	NR	NR	NR^d^	NR
				Low-dose	9605	76.0^c^	54.4					
				Warfarin	20205	77.0^c^	51.2					
Briasoulis A 2020 [9]	America	Cohort	PSM	Standard-dose	19825	NR^d^	46.8	15.1	NR^d^	NR	NR	NR^d^
				Low-dose	7922		57.0					
Brook R 2019 [40]	Australia	Cohort	NR	Standard-dose	373	69.0^c^	42.1	17.0^c^	NR	3.00^c^	NR	NR^d^
				Low-dose	285	81.0^c^	56.8			5.00^c^		
Sugrue A 2020 [41]	America	Cohort	NR	Standard-dose	7303	69.0 ± 11.8	33.7	14.4	30.5 ± 6.8	2.90 ± 1.80	NR	86.7 ± 37.4
				Low-dose	1071	71.1 ± 11.9	38.7		30.3 ± 6.6	3.50 ± 3.30		79.8 ± 40.4
Steinberg BA 2016 [42]	America	Cohort	MM	Standard-dose	5000	70.0^c^	40.1	24.0	31.7 ± 8.0	NR^d^	NR	93.2 ± 42.7
				Low-dose	541	79.0^c^	48.2		30.3 ± 7.6			66.7 ± 28.4
Almeida J 2020 [43]	Portugal	Cohort	MM	Standard-dose	160	80.0 ± 8.0	56.9	12.0	NR	4.90 ± 1.60	2.60 ± 0.90	NR
				Low-dose	167	84.0 ± 7.0	65.9			5.10 ± 1.40	2.50 ± 0.90	
Arbel R 2019 [44]	Israel	Cohort	MM	Standard-dose	5140	72.0 ± 9.0	50.0	23.0	31.0	4.37	NR	77.0
				Low-dose	3285	81.0 ± 8.0	55.0		29.0	5.05		63.0
Salameh M 2020 [45]	Israel	Cohort	MM	Standard-dose	13141	73.8 ± 8.3	48.6	60.0	NR	4.30 ± 1.60	2.70 ± 0.92	79.7 ± 28.9
				Low-dose	9885	81.4 ± 7.4	51.5			5.20 ± 1.50	2.90 ± 0.85	56.8 ± 20.8

Values are shown as the mean ± SD or *n*; PSM = propensity score matching; MM = multivariable model; BMI = body mass index; CrCl = creatinine clearance rate; NR = not reported.^a^ means characteristics are the composite of low-dose and standard-dose groups.^b^ means the CHADS_2_ score.^c^ means values are shown as the median.^d^ means values are shown as the category.

## Data Availability

All data generated or analyzed during this study are included in this published article and its supplementary information files.

## Supplementary Materials

Supplementary Materials Supplementary Table S1. Cochrane Central Register of Controlled Trials search strategy. Supplementary Table S2. Embase search strategy. Supplementary Table S3. MEDLINE search strategy. Supplementary Table S4. Detailed previous medical history of included patients. Supplementary Table S5. Detailed group contents of included studies. Supplementary Table S6. Results of quality assessment using the NEWCASTLE-OTTAWA SCALE for cohort studies. Supplementary Table S7. Results of publication bias assessment for interesting outcomes. Supplementary Table S8. Results of meta-regression analyses for low-dose NOACs versus standard-dose NOACs. Supplementary Table S9. Results of meta-regression analyses for low-dose NOACs versus warfarin. Supplementary Figure S1. Pooled stroke of low-dose NOACs versus standard-dose NOACs. Supplementary Figure S2. Pooled mortality of low-dose NOACs versus standard-dose NOACs. Supplementary Figure S3. Pooled major bleeding of low-dose NOACs versus standard-dose NOACs. Supplementary Figure S4. Pooled ICH of low-dose NOACs versus standard-dose NOACs. Supplementary Figure S5. Pooled GH of low-dose NOACs versus standard-dose NOACs. Supplementary Figure S6. Pooled adjusted stroke of low-dose NOACs versus standard-dose NOACs. Supplementary Figure S7. Pooled adjusted mortality of low-dose NOACs versus standard-dose NOACs. Supplementary Figure S8. Pooled adjusted major bleeding of low-dose NOACs versus standard-dose NOACs. Supplementary Figure S9. Pooled adjusted ICH of low-dose NOACs versus standard-dose NOACs. Supplementary Figure S10. Result of meta-regression between major bleeding and mean age for low-dose NOACs versus standard-dose NOACs. Supplementary Figure S11. Result of meta-regression between ICH and mean age for low-dose NOACs versus standard-dose NOACs. Supplementary Figure S12. Result of meta-regression between ICH and female for low-dose NOACs versus standard-dose NOACs. Supplementary Figure S13. Pooled major bleeding of low-dose NOACs versus standard-dose NOACs stratified by mean age. Supplementary Figure S14. Pooled ICH of low-dose NOACs versus standard-dose NOACs stratified by mean age. Supplementary Figure S15. Pooled stroke of low-dose NOACs versus warfarin. Supplementary Figure S16. Pooled mortality of low-dose NOACs versus warfarin. Supplementary Figure S17. Pooled major bleeding of low-dose NOACs versus warfarin. Supplementary Figure S18. Pooled ICH of low-dose NOACs versus warfarin. Supplementary Figure S19. Pooled GH of low-dose NOACs versus warfarin. Supplementary Figure S20. Pooled adjusted stroke of low-dose NOACs versus warfarin. Supplementary Figure S21. Pooled adjusted major bleeding of low-dose NOACs versus warfarin.
