# Supplementary material for: Real-World Comparisons of Low-Dose NOACs versus Standard-Dose NOACs or Warfarin on Efficacy and Safety in Patients with AF: A Meta-Analysis
Source: Cardiol Res Pract. 2022 Mar 7;2022:4713826. doi: 10.1155/2022/4713826 (PMC9017587; doi:10.1155/2022/4713826)
Supplement: Supplementary Materials — Supplementary Table S1. Cochrane Central Register of Controlled Trials search strategy. Supplementary Table S2. Embase search strategy. Supplementary Table S3. MEDLINE search strategy. Supplementary Table S4. Detailed previous medical history of included patients. Supplementary Table S5. Detailed group contents of included studies. Supplementary Table S6. Results of quality assessment using the NEWCASTLE-OTTAWA SCALE for cohort studies. Supplementary Table S7. Results of publication bias assessment for interesting outcomes. Supplementary Table S8. Results of meta-regression analyses for low-dose NOACs versus standard-dose NOACs. Supplementary Table S9. Results of meta-regression analyses for low-dose NOACs versus warfarin. Supplementary Figure S1. Pooled stroke of low-dose NOACs versus standard-dose NOACs. Supplementary Figure S2. Pooled mortality of low-dose NOACs versus standard-dose NOACs. Supplementary Figure S3. Pooled major bleeding of low-dose NOACs versus standard-dose NOACs. Supplementary Figure S4. Pooled ICH of low-dose NOACs versus standard-dose NOACs. Supplementary Figure S5. Pooled GH of low-dose NOACs versus standard-dose NOACs. Supplementary Figure S6. Pooled adjusted stroke of low-dose NOACs versus standard-dose NOACs. Supplementary Figure S7. Pooled adjusted mortality of low-dose NOACs versus standard-dose NOACs. Supplementary Figure S8. Pooled adjusted major bleeding of low-dose NOACs versus standard-dose NOACs. Supplementary Figure S9. Pooled adjusted ICH of low-dose NOACs versus standard-dose NOACs. Supplementary Figure S10. Result of meta-regression between major bleeding and mean age for low-dose NOACs versus standard-dose NOACs. Supplementary Figure S11. Result of meta-regression between ICH and mean age for low-dose NOACs versus standard-dose NOACs. Supplementary Figure S12. Result of meta-regression between ICH and female for low-dose NOACs versus standard-dose NOACs. Supplementary Figure S13. Pooled major bleeding of low-dose NOACs versus [file 4713826.f1.docx]

**Supplementary Materials**

**Contents**

[Table S1. Cochrane Central Register of Controlled Trials search strategy 2](#_Toc88953884)

[Table S2. Embase search strategy 2](#_Toc88953885)

[Table S3. MEDLINE search strategy 3](#_Toc88953886)

[Table S4. Detailed previous medical history of included patients 5](#_Toc88953887)

[Table S5. Detailed group contents of included studies 9](#_Toc88953888)

[Table S6. Results of quality assessment using the NEWCASTLE-OTTAWA SCALE for cohort studies 13](#_Toc88953889)

[Table S7. Results of publication bias assessment for interesting outcomes 17](#_Toc88953890)

[Table S8. Results of meta-regression analyses for low-dose NOACs versus standard-dose NOACs 17](#_Toc88953891)

[Table S9. Results of meta-regression analyses for low-dose NOACs versus warfarin 18](#_Toc88953892)

[Figure S1. Pooled ischemic stroke of low-dose NOACs versus standard-dose NOACs 19](#_Toc88953893)

[Figure S2. Pooled mortality of low-dose NOACs versus standard-dose NOACs 20](#_Toc88953894)

[Figure S3. Pooled major bleeding of low-dose NOACs versus standard-dose NOACs 21](#_Toc88953895)

[Figure S4. Pooled ICH of low-dose NOACs versus standard-dose NOACs 22](#_Toc88953896)

[Figure S5. Pooled GH of low-dose NOACs versus standard-dose NOACs 23](#_Toc88953897)

[Figure S6. Pooled adjusted ischemic stroke of low-dose NOACs versus standard-dose NOACs 24](#_Toc88953898)

[Figure S7. Pooled adjusted mortality of low-dose NOACs versus standard-dose NOACs 25](#_Toc88953899)

[Figure S8. Pooled adjusted major bleeding of low-dose NOACs versus standard-dose NOACs 26](#_Toc88953900)

[Figure S9. Pooled adjusted ICH of low-dose NOACs versus standard-dose NOACs 27](#_Toc88953901)

[Figure S10. Result of meta-regression between major bleeding and mean age for low-dose NOACs versus standard-dose NOACs 28](#_Toc88953902)

[Figure S11. Result of meta-regression between ICH and mean age for low-dose NOACs versus standard-dose NOACs 29](#_Toc88953903)

[Figure S12. Result of meta-regression between ICH and female for low-dose NOACs versus standard-dose NOACs 30](#_Toc88953904)

[Figure S13. Pooled major bleeding of low-dose NOACs versus standard-dose NOACs stratified by mean age 31](#_Toc88953905)

[Figure S14. Pooled ICH of low-dose NOACs versus standard-dose NOACs stratified by mean age 32](#_Toc88953906)

[Figure S15. Pooled ischemic stroke of low-dose NOACs versus warfarin 33](#_Toc88953907)

[Figure S16. Pooled mortality of low-dose NOACs versus warfarin 34](#_Toc88953908)

[Figure S17. Pooled major bleeding of low-dose NOACs versus warfarin 35](#_Toc88953909)

[Figure S18. Pooled ICH of low-dose NOACs versus warfarin 36](#_Toc88953910)

[Figure S19. Pooled GH of low-dose NOACs versus warfarin 37](#_Toc88953911)

[Figure S20. Pooled adjusted ischemic stroke of low-dose NOACs versus warfarin 38](#_Toc88953912)

[Figure S21. Pooled adjusted major bleeding of low-dose NOACs versus warfarin 39](#_Toc88953913)

Table S1. Cochrane Central Register of Controlled Trials search strategy

| Cochrane Central Register of Controlled Trials |
| --- |
| #1 atrial fibrillat* OR atrium fibrillat* OR atrial fibrillation in Title Abstract Keyword |
| #2 warfarin* OR acenocoumarol OR dicoumarol OR coumadin OR diphenadione OR 'vitamin k antagonist*' OR vka OR 'factor xa inhibitor*' OR antithrombin* OR anticoagul* OR xarelto OR apixaban OR eliquis OR 'dabigatran etexilate' OR edoxaban OR savaysa OR rivaroxaban OR dabigatran OR 'target specific oral anticoagulant*' OR 'target-specific oral anticoagulant*' OR tsoac* OR 'new oral anticoagulant*' OR 'novel oral anticoagulant*' OR noac* OR 'direct-acting oral anticoagulant*' OR 'direct acting oral anticoagulant*' OR 'direct oral anticoagulant*' OR doac in Title Abstract Keyword |
| #3 'low dose' OR 'micro dose' OR 'off label' OR underdosing OR underdose OR underdosed OR 'reduced dose' in All Text |
| #4 #1 and #2 and #3 |

Table S2. Embase search strategy

| Embase |  |
| --- | --- |
| 1. 'atrial fibrillat*':ab,ti OR 'atrium fibrillat*':ab,ti OR 'atrial fibrillation':ab,ti |  |
| 2. warfarin*:ab,ti OR acenocoumarol:ab,ti OR dicoumarol:ab,ti OR coumadin:ab,ti OR diphenadione:ab,ti OR 'vitamin k antagonist*':ab,ti OR vka:ab,ti OR 'factor xa inhibitor*':ab,ti OR antithrombin*:ab,ti OR anticoagul*:ab,ti OR xarelto:ab,ti OR apixaban:ab,ti OR eliquis:ab,ti OR 'dabigatran etexilate':ab,ti OR edoxaban:ab,ti OR savaysa:ab,ti OR rivaroxaban:ab,ti OR dabigatran:ab,ti OR 'target specific oral anticoagulant*':ab,ti OR 'target-specific oral anticoagulant*':ab,ti OR tsoac*:ab,ti OR 'new oral anticoagulant*':ab,ti OR 'novel oral anticoagulant*':ab,ti OR noac*:ab,ti OR 'direct-acting oral anticoagulant*':ab,ti OR 'direct acting oral anticoagulant*':ab,ti OR 'direct oral anticoagulant*':ab,ti OR doac:ab,ti |  |
| 3. 'low dose':ab,ti OR 'micro dose':ab,ti OR 'off label':ab,ti OR underdosing:ab,ti OR underdose:ab,ti OR underdosed:ab,ti OR 'reduced dose':ab,ti |  |
| 4. 1 and 2 and 3 |  |

Table S3. MEDLINE search strategy

| MEDLINE |  |
| --- | --- |
| 1. atrial fibrillat*[Title/Abstract] OR atrium fibrillat*[Title/Abstract] OR atrial fibrillation[Title/Abstract] |  |
| 2. warfarin*[Title/Abstract] OR acenocoumarol[Title/Abstract] OR dicoumarol[Title/Abstract] OR coumadin[Title/Abstract] OR diphenadione[Title/Abstract] OR 'vitamin k antagonist*'[Title/Abstract] OR vka[Title/Abstract] OR 'factor xa inhibitor*'[Title/Abstract] OR antithrombin*[Title/Abstract] OR anticoagul*[Title/Abstract] OR xarelto[Title/Abstract] OR apixaban[Title/Abstract] OR eliquis[Title/Abstract] OR 'dabigatran etexilate'[Title/Abstract] OR edoxaban[Title/Abstract] OR savaysa[Title/Abstract] OR rivaroxaban[Title/Abstract] OR dabigatran[Title/Abstract] OR 'target specific oral anticoagulant*'[Title/Abstract] OR 'target-specific oral anticoagulant*'[Title/Abstract] OR tsoac*[Title/Abstract] OR 'new oral anticoagulant*'[Title/Abstract] OR 'novel oral anticoagulant*'[Title/Abstract] OR noac*[Title/Abstract] OR 'direct-acting oral anticoagulant*'[Title/Abstract] OR 'direct acting oral anticoagulant*'[Title/Abstract] OR 'direct oral anticoagulant*'[Title/Abstract] OR doac[Title/Abstract] |  |
| 3. low dose' OR 'micro dose' OR 'off label' OR underdosing OR underdose OR underdosed OR 'reduced dose' |  |
| 4. 1 and 2 and 3 |  |

Table S4. Detailed previous medical history of included patients

| Author, year | Group | Previous medical history (%) | | | | | |
| --- | --- | --- | --- | --- | --- | --- | --- |
|  |  | Hypertension | Diabetes | Heart failure | Vascular disease | Stroke/TIA | Major bleeding |
| Murata N 2019 | Standard-dose | 68.1 | 22.3 | 16.4 | 9.9 | 9.5 | 0.5 |
|  | Low-dose | 71.3 | 22.2 | 17.9 | 14.4 | 7.6 | 1.4 |
| Wakamatsu Y 2020 | Standard-dose | 61.3 | 20.4 | 15.2 | 9.8 | 11.9 | 1.5 |
|  | Low-dose | 62.5 | 17.6 | 17.1 | 13.9 | 12.5 | 2.3 |
| Ohno J 2021 | Standard-dose | 71.0 | 28.8 | 18.3 | 6.2 | 14.9 | NR |
|  | Low-dose | 71.6 | 27.2 | 17.8 | 10.2 | 22.5 |  |
| Lee HF 2018 | Low-dose | 86.0 | 39.0 | 14.0 | NR | 22.0 | 2.5 |
|  | warfarin | 86.0 | 39.0 | 14.0 |  | 21.0 | 2.0 |
| Yu HT 2018 | Low-dose | 94.0 | 34.6 | 66.9 | 32.8 | 40.6 | NR |
|  | warfarin | 94.6 | 34.3 | 67.5 | 32.6 | 40.4 |  |
| Chan YH 2018 | Standard-dose^a^ | 87.0 | 41.0 | 13.0 | NR | 23.0 | 2.0 |
|  | Low-dose^a^ |  |  |  |  |  |  |
|  | warfarin | 87.0 | 40.0 | 13.0 |  | 23.0 | 2.0 |
| Chang HK 2016 | Standard-dose^a^ | 72.3 | 25.7 | 18.2 | NR | 45.9 | NR |
|  | Low-dose^a^ |  |  |  |  |  |  |
|  | warfarin | 75.2 | 49.5 | 20.0 |  | 37.9 |  |
| Akagi Y 2019 | Standard-dose^a^ | 60.1 | 19.7 | 19.0 | NR | 26.2 | NR |
|  | Low-dose^a^ |  |  |  |  |  |  |
| Yu HT 2020 | Standard-dose | 94.5 | 31.4 | 60.4 | 27.9 | 46.6 | NR |
|  | Low-dose | 95.3 | 32.3 | 60.4 | 29.7 | 41.6 |  |
| Cho MS 2019 | Low-dose | 87.8 | 45.5 | 20.5 | 11.5 | 21.1 | NR |
|  | warfarin | 86.7 | 48.4 | 22.8 | 12.8 | 27.3 |  |
| Jeong HK 2019 | Low-dose | 53.5 | 24.1 | 5.7 | NR | 29.2 | NR |
|  | warfarin | 54.7 | 22.3 | 5.1 |  | 29.2 |  |
| Kohsaka S 2020 | Low-dose | 54.9 | 30.0 | 37.1 | NR | 21.2 | NR |
|  | warfarin | 55.9 | 30.4 | 37.5 |  | 21.4 |  |
| Kohsaka S 2017 | Low-dose | 53.8 | 28.9 | 35.3 | 6.6 | 22.3 | NR |
|  | warfarin | 54.0 | 28.2 | 35.4 | 6.2 | 22.6 |  |
| Lai CL 2018 | Low-dose | 51.1 | 16.9 | 25.3 | 4.2 | 16.3 | NR |
|  | warfarin | 50.3 | 15.4 | 29.6 | 4.1 | 11.6 |  |
| Lee SR 2019 | Standard-dose | 72.0 | 21.5 | 30.2 | NR | NR | NR |
|  | Low-dose | 73.1 | 21.1 | 31.2 |  |  |  |
|  | warfarin | 72.3 | 22.3 | 32.4 |  |  |  |
| Chan YH 2019 | Low-dose | 84.1 | 38.1 | 11.1 | NR | 15.2 | NR |
|  | warfarin | 84.5 | 38.6 | 10.8 |  | 15.0 |  |
| Alcusky M 2018 | Standard-dose | 85.6 | 30.7 | 20.6 | NR | NR | NR |
|  | Low-dose | 88.1 | 26.8 | 23.5 |  |  |  |
| Bouget J 2020 | Standard-dose | NR | 11.8 | NR | NR | NR | NR |
|  | Low-dose |  | 10.6 |  |  |  |  |
|  | warfarin |  | 15.9 |  |  |  |  |
| Briasoulis A 2020 | Standard-dose | 92.3 | 47.9 | 25.6 | NR | 22.8 | 22.8 |
|  | Low-dose | 97.5 | 50.9 | 38.0 |  | 27.9 | 26.6 |
| Brook R 2019 | Standard-dose | NR | NR | NR | NR | NR | NR |
|  | Low-dose |  |  |  |  |  |  |
| Sugrue A 2020 | Standard-dose | 67.2 | 23.6 | 32.0 | 8.9 | NR | NR |
|  | Low-dose | 71.7 | 30.6 | 35.9 | 10.6 |  |  |
| Steinberg BA 2016 | Standard-dose | NR | NR | 20.0 | 11.5 | NR | NR |
|  | Low-dose |  |  | 25.7 | 14.6 |  |  |
| Almeida J 2020 | Standard-dose | 83.1 | 44.0 | 63.8 | 19.5 | 26.3 | 2.4 |
|  | Low-dose | 85.5 | 36.4 | 70.5 | 15.8 | 23.0 | 5.0 |
| Arbel R 2019 | Standard-dose | 95.0 | 63.0 | 24.0 | 15.0 | NR | NR |
|  | Low-dose | 96.0 | 55.0 | 33.0 | 20.0 |  |  |
| Salameh M 2020 | Standard-dose | 87.2 | 57.3 | 24.7 | 9.7 | 30.7 | NR |
|  | Low-dose | 92.3 | 47.6 | 37.5 | 14.2 | 38.3 |  |

TIA = transient ischemic attack; NR = not reported.

^a^ means characteristics are the composite of low-dose and standard-dose groups.

Table S5. Detailed group contents of included studies

| Author, year | Standard-dose | Low-dose |
| --- | --- | --- |
| Murata N 2019 | dabigatran  rivaroxaban  apixaban  edoxaban | dabigatran 110 mg (b.i.d.) rivaroxaban 10 mg (q.d.) apixaban 2.5 mg (b.i.d.)  edoxaban 30 mg (q.d.) |
| Wakamatsu Y 2020 | dabigatran  rivaroxaban  apixaban  edoxaban | dabigatran 110 mg (b.i.d.) rivaroxaban 10 mg (q.d.) apixaban 2.5 mg (b.i.d.)  edoxaban 30 mg (q.d.) |
| Ohno J 2021 | dabigatran  rivaroxaban  apixaban  edoxaban | dabigatran 110 mg (b.i.d.) rivaroxaban 10 mg (q.d.) apixaban 2.5 mg (b.i.d.)  edoxaban 30 mg (q.d.) |
| Akagi Y 2019 | dabigatran | dabigatran 110 mg (b.i.d.) |
| Yu HT 2020 | dabigatran  rivaroxaban  apixaban  edoxaban | dabigatran 110 mg (b.i.d.) rivaroxaban 15/10 mg (q.d.) apixaban 2.5 mg (b.i.d.)  edoxaban 30 mg (q.d.) |
| Chan YH 2018 | dabigatran  rivaroxaban  apixaban | dabigatran 110 mg (b.i.d.) rivaroxaban 15/10 mg (q.d.) apixaban 2.5 mg (b.i.d.) |
| Chang HK 2016 | dabigatran  rivaroxaban  warfarin | dabigatran 110 mg (b.i.d.) rivaroxaban 15 mg (q.d.) |
| Lee SR 2019 | rivaroxaban  warfarin | rivaroxaban 15 mg (q.d.) |
| Yu HT 2018 | warfarin | edoxaban 30 mg (q.d.) |
| Lee HF 2018 | warfarin | rivaroxaban 15/10 mg (q.d.) |
| Cho MS 2019 | warfarin | dabigatran 110 mg (b.i.d.) rivaroxaban 15/10 mg (q.d.) apixaban 2.5 mg (b.i.d.) |
| Jeong HK 2019 | warfarin | rivaroxaban 15 mg (q.d.) |
| Kohsaka S 2017 | warfarin | rivaroxaban 15/10 mg (q.d.) |
| Kohsaka S 2020 | warfarin | rivaroxaban 15/10 mg (q.d.) |
| Lai CL 2018 | warfarin | dabigatran 110 mg (b.i.d.) |
| Chan YH 2019 | warfarin | dabigatran 110 mg (b.i.d.) rivaroxaban 15/10 mg (q.d.) apixaban 2.5 mg (b.i.d.)  edoxaban 30/15 mg (q.d.) |
| Alcusky M 2018 | dabigatran rivaroxaban | dabigatran 75 mg (b.i.d.)  rivaroxaban 15/10 mg (q.d.) |
| Bouget J 2020 | dabigatran rivaroxaban apixaban  warfarin | dabigatran 110 mg (b.i.d.)  rivaroxaban 10 mg (q.d.)  apixaban 2.5 mg (b.i.d.) |
| Briasoulis A 2020 | dabigatran rivaroxaban | dabigatran 75 mg (b.i.d.)  rivaroxaban 15 mg (q.d.) |
| Brook R 2019 | dabigatran rivaroxaban apixaban | dabigatran 110 mg (b.i.d.)  rivaroxaban 15 mg (q.d.)  apixaban 2.5 mg (b.i.d.) |
| Sugrue A 2020 | dabigatran rivaroxaban apixaban | dabigatran 110 mg (b.i.d.)  rivaroxaban 15/10 mg (q.d.)  apixaban 2.5 mg (b.i.d.) |
| Steinberg BA 2016 | dabigatran rivaroxaban apixaban | dabigatran 110 mg (b.i.d.)  rivaroxaban 15/10 mg (q.d.)  apixaban 2.5 mg (b.i.d.) |
| Almeida J 2020 | dabigatran rivaroxaban apixaban | dabigatran 75 mg (b.i.d.)  rivaroxaban 15 mg (q.d.)  apixaban 2.5 mg (b.i.d.) |
| Arbel R 2019 | NR | NR |
| Salameh M 2020 | apixaban | apixaban 2.5 mg (b.i.d.) |

Table S6. Results of quality assessment using the NEWCASTLE-OTTAWA SCALE for cohort studies

| Author, year | Selection | | | | Comparability | Outcome | | |
| --- | --- | --- | --- | --- | --- | --- | --- | --- |
|  | Representati-  veness of the exposed cohort | Selection of the non-exposed cohort | Ascertainment of exposure | Demonstration that outcome of interest was not present at start of study | Comparability of cohorts on the basis of the design or analysis | Assessment of outcome | Was follow-up long enough for outcomes to occur | Adequacy of follow up of cohorts |
| Murata N | **★** | ★ | ★ | ★ | ★★ | ★ | ★ | ★ |
| 2019 |  |  |  |  |  |  |  |  |
| Wakamatsu Y | ★ | ★ | ★ | ★ | ☆☆ | ★ | ★ | ☆ |
| 2020 |  |  |  |  |  |  |  |  |
| Ohno J | ★ | ★ | ★ | ★ | ★★ | ★ | ★ | ☆ |
| 2021 |  |  |  |  |  |  |  |  |
| Lee HF | ★ | ★ | ★ | ★ | ★★ | ★ | ☆ | ☆ |
| 2018 |  |  |  |  |  |  |  |  |
| Chang HK | ★ | ★ | ★ | ★ | ☆☆ | ★ | ★ | ☆ |
| 2016 |  |  |  |  |  |  |  |  |
| Akagi Y | ★ | ★ | ★ | ★ | ☆☆ | ★ | ☆ | ☆ |
| 2019 |  |  |  |  |  |  |  |  |
| Yu HT | ★ | ★ | ★ | ★ | ★★ | ★ | ★ | ☆ |
| 2020 |  |  |  |  |  |  |  |  |
| Yu HT | ★ | ★ | ★ | ★ | ★★ | ★ | ★ | ☆ |
| 2018 |  |  |  |  |  |  |  |  |
| Cho MS | ★ | ★ | ★ | ★ | ★★ | ★ | ★ | ☆ |
| 2019 |  |  |  |  |  |  |  |  |
| Jeong HK | ★ | ★ | ★ | ★ | ★★ | ★ | ★ | ☆ |
| 2019 |  |  |  |  |  |  |  |  |
| Kohsaka S | ★ | ★ | ★ | ★ | ★★ | ★ | ★ | ☆ |
| 2020 |  |  |  |  |  |  |  |  |
| Kohsaka S | ★ | ★ | ★ | ★ | ★★ | ★ | ☆ | ☆ |
| 2017 |  |  |  |  |  |  |  |  |
| Lai CL | ★ | ★ | ★ | ★ | ★★ | ★ | ★ | ☆ |
| 2018 |  |  |  |  |  |  |  |  |
| Lee SR | ★ | ★ | ★ | ★ | ★★ | ★ | ★ | ☆ |
| 2019 |  |  |  |  |  |  |  |  |
| Chan YH | ★ | ★ | ★ | ★ | ★★ | ★ | ★ | ☆ |
| 2018 |  |  |  |  |  |  |  |  |
| Chan YH | ★ | ★ | ★ | ★ | ★★ | ★ | ★ | ☆ |
| 2019 |  |  |  |  |  |  |  |  |
| Alcusky M | ★ | ★ | ★ | ★ | ☆☆ | ★ | ★ | ★ |
| 2018 |  |  |  |  |  |  |  |  |
| Bouget J | ★ | ★ | ★ | ★ | ☆☆ | ★ | ★ | ☆ |
| 2020 |  |  |  |  |  |  |  |  |
| Briasoulis A | ★ | ★ | ★ | ★ | ★★ | ★ | ★ | ☆ |
| 2020 |  |  |  |  |  |  |  |  |
| Brook R | ★ | ★ | ★ | ★ | ☆☆ | ★ | ★ | ☆ |
| 2019 |  |  |  |  |  |  |  |  |
| Sugrue A | ★ | ★ | ★ | ★ | ☆☆ | ★ | ★ | ☆ |
| 2020 |  |  |  |  |  |  |  |  |
| Steinberg BA | ★ | ★ | ★ | ★ | ★★ | ★ | ★ | ☆ |
| 2016 |  |  |  |  |  |  |  |  |
| Almeida J | ★ | ★ | ★ | ★ | ★★ | ★ | ★ | ☆ |
| 2020 |  |  |  |  |  |  |  |  |
| Arbel R | ★ | ★ | ★ | ★ | ★★ | ★ | ★ | ☆ |
| 2019 |  |  |  |  |  |  |  |  |
| Salameh M | ★ | ★ | ★ | ★ | ★★ | ★ | ★ | ☆ |
| 2020 |  |  |  |  |  |  |  |  |

Table S7. Results of publication bias assessment for interesting outcomes

| Comparison | Outcomes | | | | | | | | | |
| --- | --- | --- | --- | --- | --- | --- | --- | --- | --- | --- |
|  | Ischemic stroke | | Mortality | | Major bleeding | | ICH | | GH | |
|  | Begg's test | Egger's test | Begg's test | Egger's test | Begg's test | Egger's test | Begg's test | Egger's test | Begg's test | Egger's test |
| Low-dose NOACs versus standard-dose NOACs | 1.000 | 0.533 | 0.436 | 0.371 | 0.945 | 0.898 | 1.000 | 0.833 | 0.548 | 0.225 |
| Low-dose NOACs versus warfarin | 0.230 | 0.235 | 0.764 | 0.942 | 0.174 | 0.221 | 0.452 | 0.035 | 0.260 | 0.279 |

ICH = intracranial haemorrhage; GH = gastrointestinal haemorrhage.

Table S8. Results of meta-regression analyses for low-dose NOACs versus standard-dose NOACs

| Low-dose NOACs versus standard-dose NOACs | | | | | |
| --- | --- | --- | --- | --- | --- |
| Variables | Ischemic stroke (*P* value) | Mortality (*P* value) | Major bleeding (*P* value) | ICH (*P* value) | GH (*P* value) |
| Mean age | 0.271 | 0.256 | 0.010 | 0.046 | 0.053 |
| Female | 0.673 | 0.558 | 0.174 | 0.035 | 0.486 |
| BMI | 0.770 | 0.839 | 0.937 | 0.635 | NA |
| CHA_2_DS_2_-VASc | 0.218 | 0.772 | 0.328 | 0.085 | 0.781 |
| HAS-BLED | 0.308 | 0.798 | 0.223 | 0.144 | NA |
| CrCl | 0.380 | 0.791 | 0.113 | 0.050 | NA |

BMI = body mass index; CrCl = creatinine clearance rate; NA = not available; ICH = intracranial haemorrhage; GH = gastrointestinal haemorrhage.

Table S9. Results of meta-regression analyses for low-dose NOACs versus warfarin

| Low-dose NOACs versus warfarin | | | | | |
| --- | --- | --- | --- | --- | --- |
| Variables | Ischemic stroke (*P* value) | Mortality (*P* value) | Major bleeding (*P* value) | ICH (*P* value) | GH (*P* value) |
| Mean age | 0.780 | 0.416 | 0.998 | 0.302 | 0.990 |
| Female | 0.544 | 0.883 | 0.996 | 0.104 | 0.759 |
| BMI | 0.345 | NA | 0.999 | NA | NA |
| CHA_2_DS_2_-VASc | 0.141 | 0.144 | 0.999 | 0.808 | 0.545 |
| HAS-BLED | 0.925 | NA | 0.999 | NA | NA |
| CrCl | NA | NA | NA | NA | NA |

BMI = body mass index; CrCl = creatinine clearance rate; NA = not available; ICH = intracranial haemorrhage; GH = gastrointestinal haemorrhage.


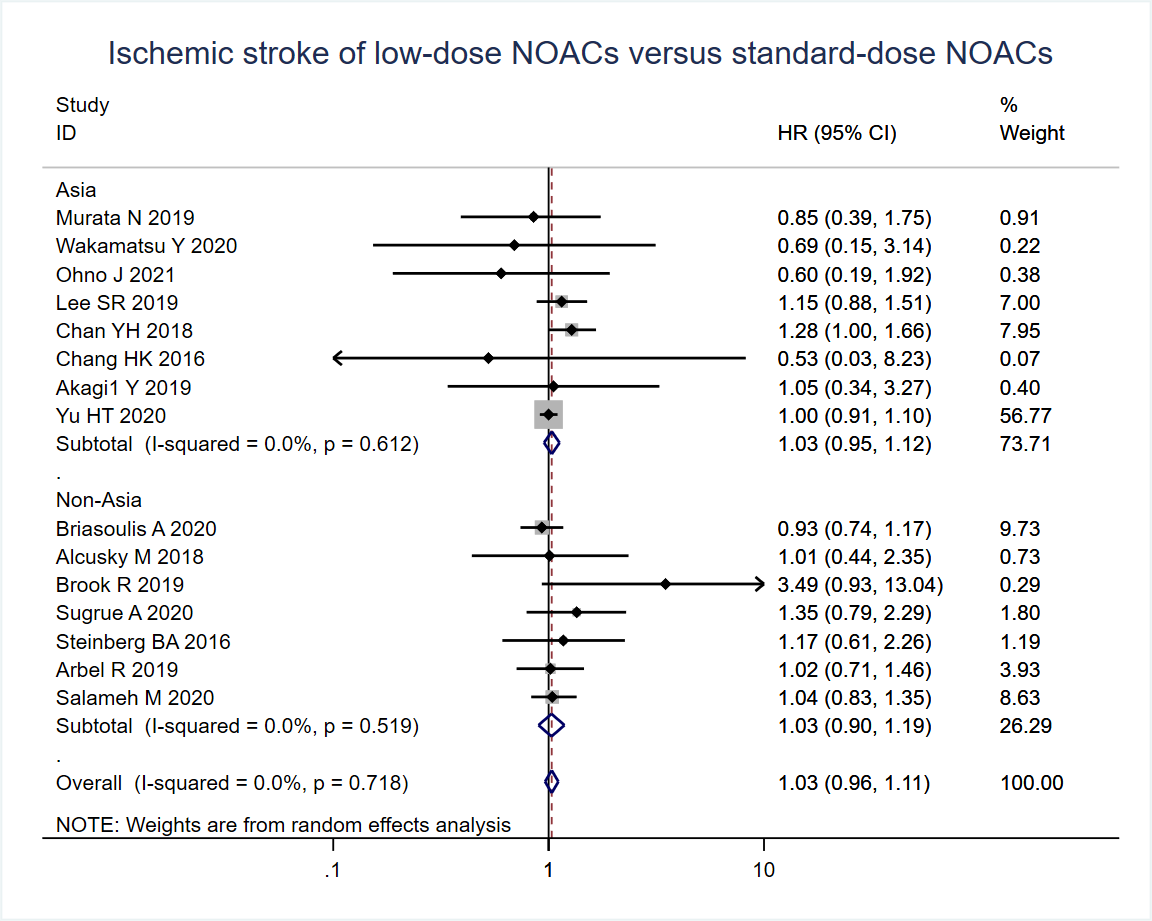


Figure S1. Pooled ischemic stroke of low-dose NOACs versus standard-dose NOACs

HR = hazard ratio.


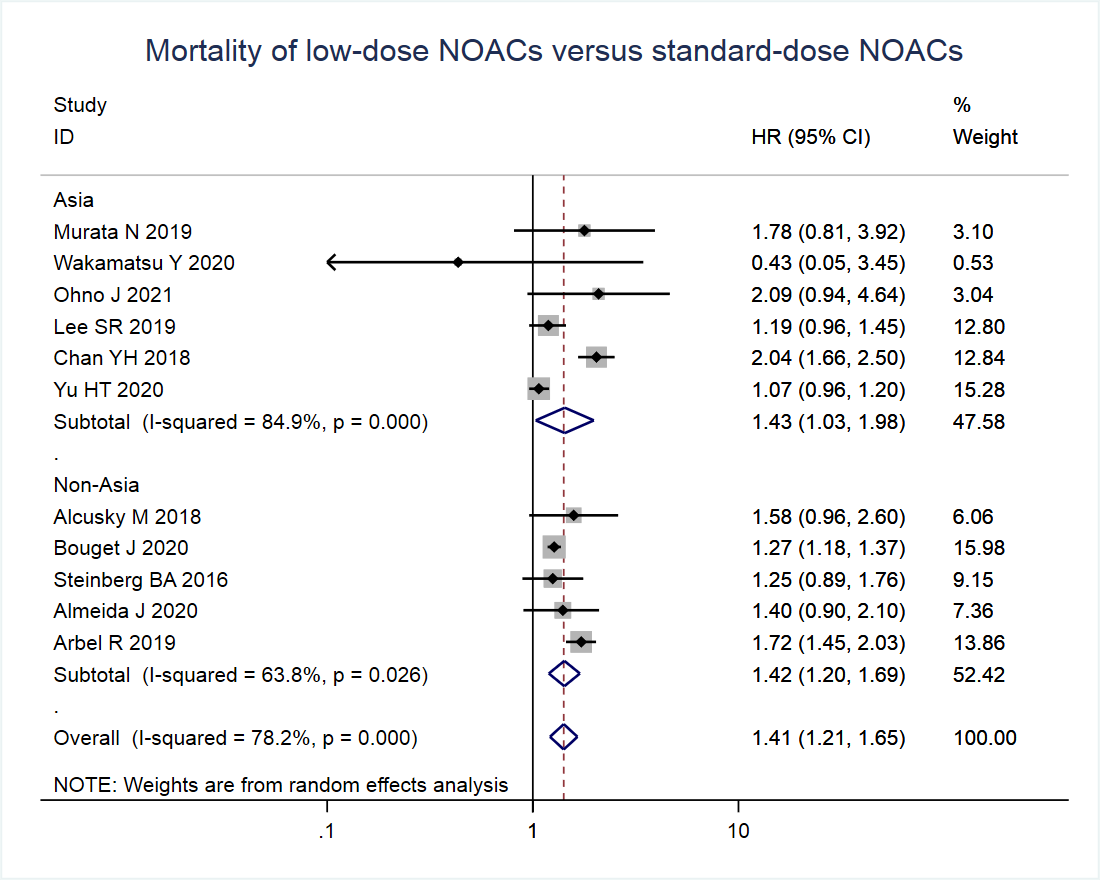
Figure S2. Pooled mortality of low-dose NOACs versus standard-dose NOACs

HR = hazard ratio.


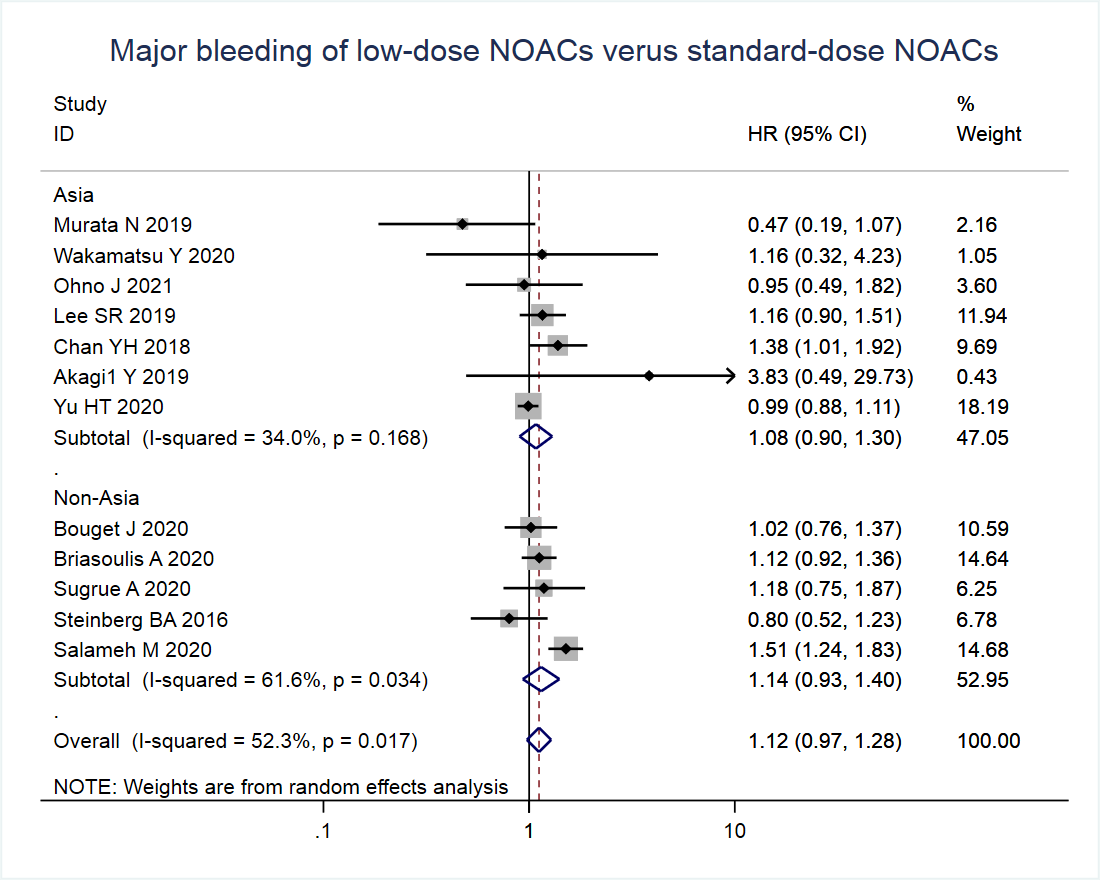
Figure S3. Pooled major bleeding of low-dose NOACs versus standard-dose NOACs

HR = hazard ratio.


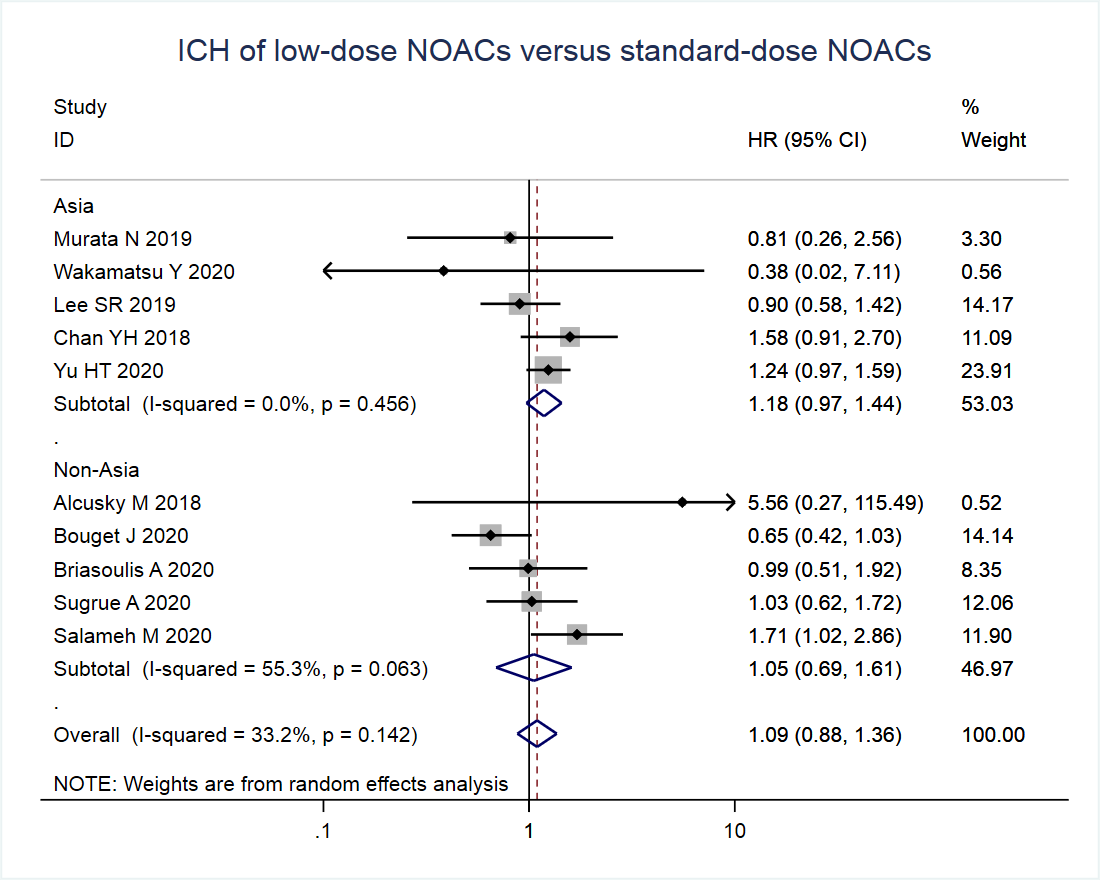
Figure S4. Pooled ICH of low-dose NOACs versus standard-dose NOACs

ICH = intracranial haemorrhage; HR = hazard ratio.


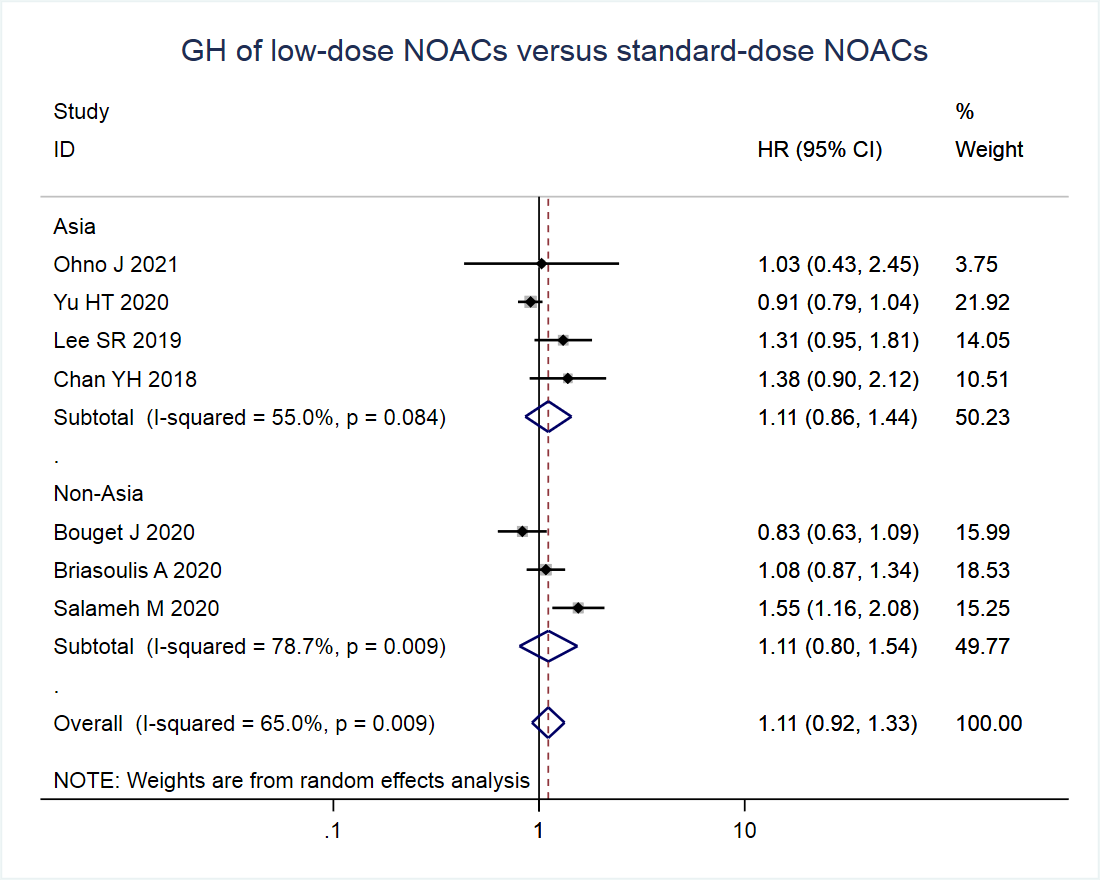
Figure S5. Pooled GH of low-dose NOACs versus standard-dose NOACs

GH = gastrointestinal haemorrhage; HR = hazard ratio.


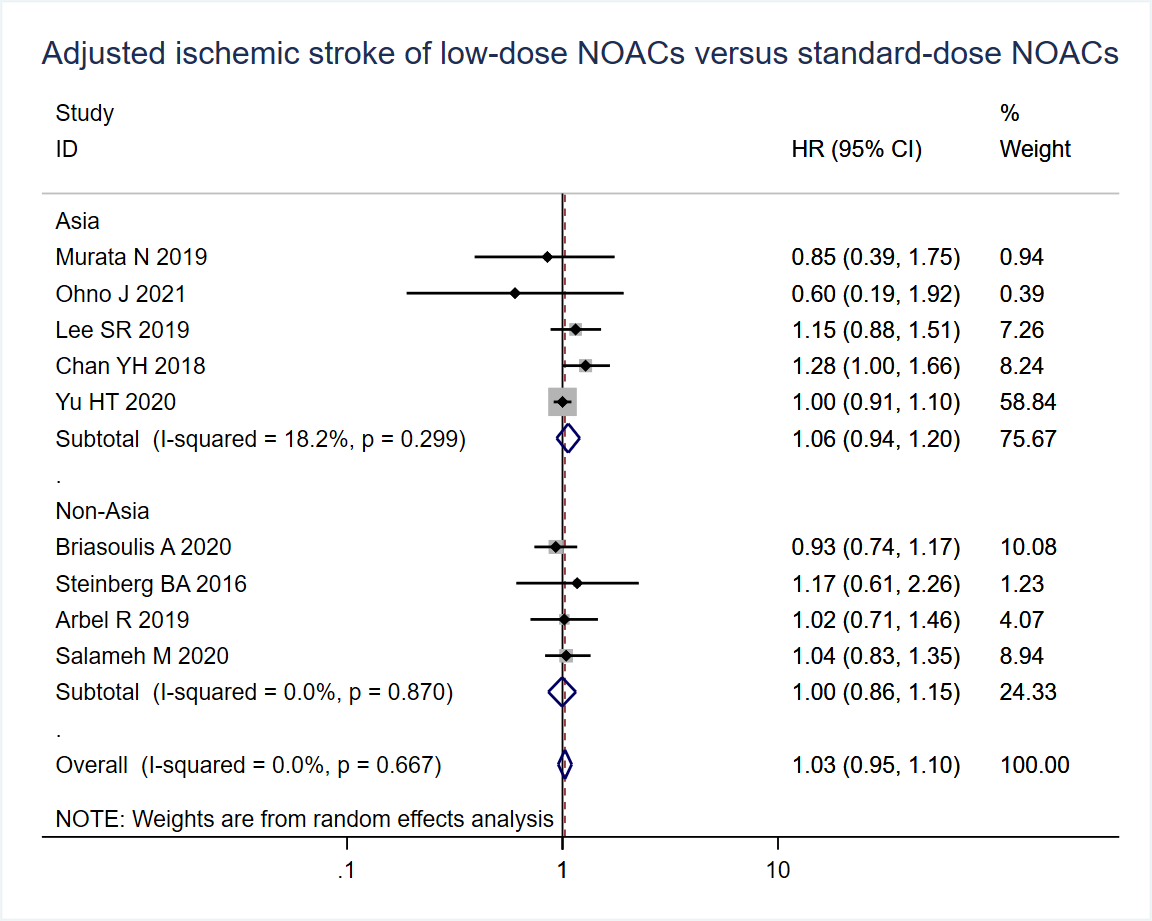


Figure S6. Pooled adjusted ischemic stroke of low-dose NOACs versus standard-dose NOACs

HR = hazard ratio.


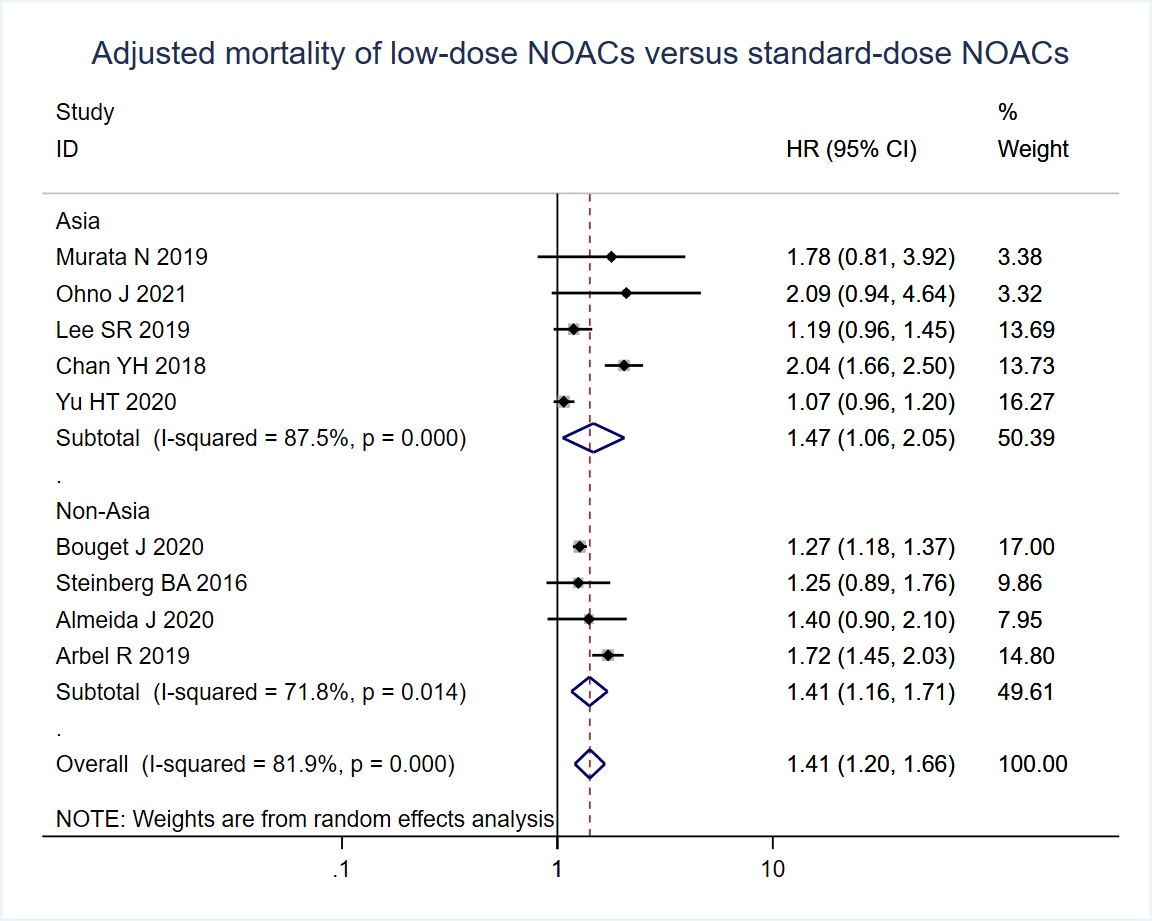


Figure S7. Pooled adjusted mortality of low-dose NOACs versus standard-dose NOACs

HR = hazard ratio.


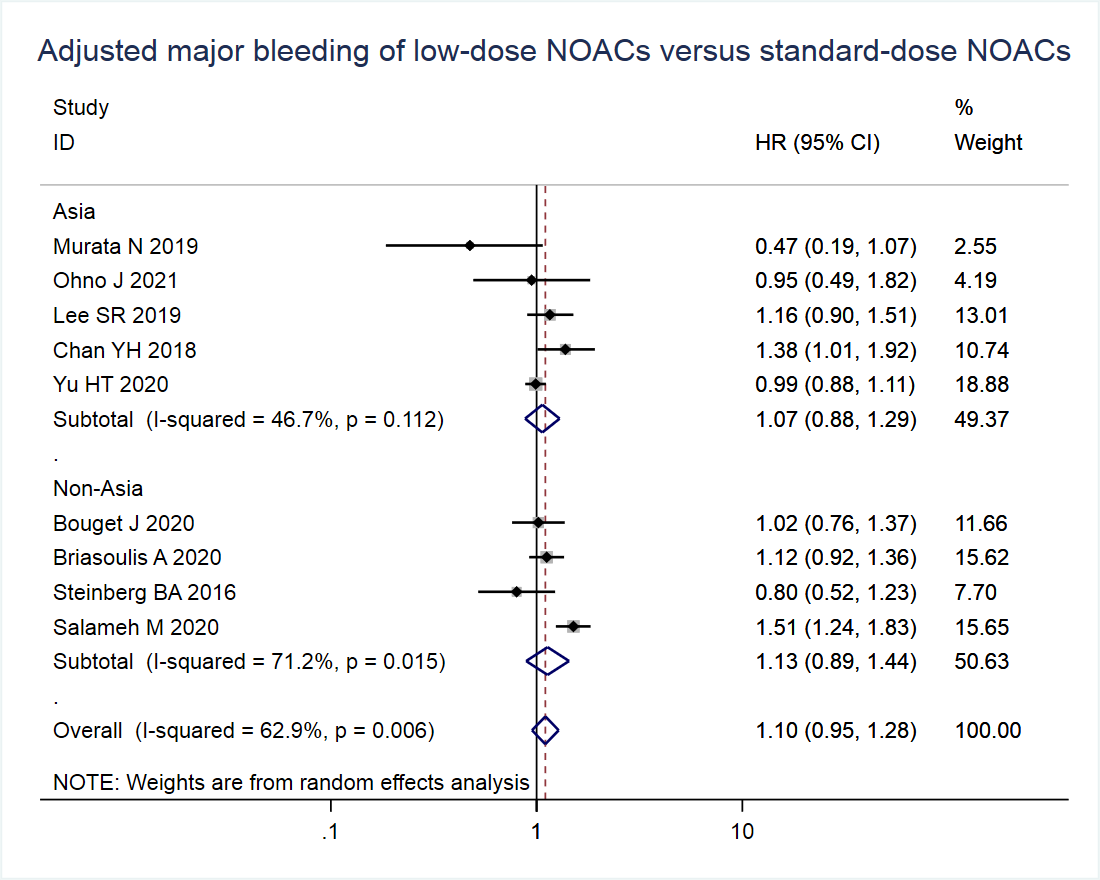
Figure S8. Pooled adjusted major bleeding of low-dose NOACs versus standard-dose NOACs

HR = hazard ratio.


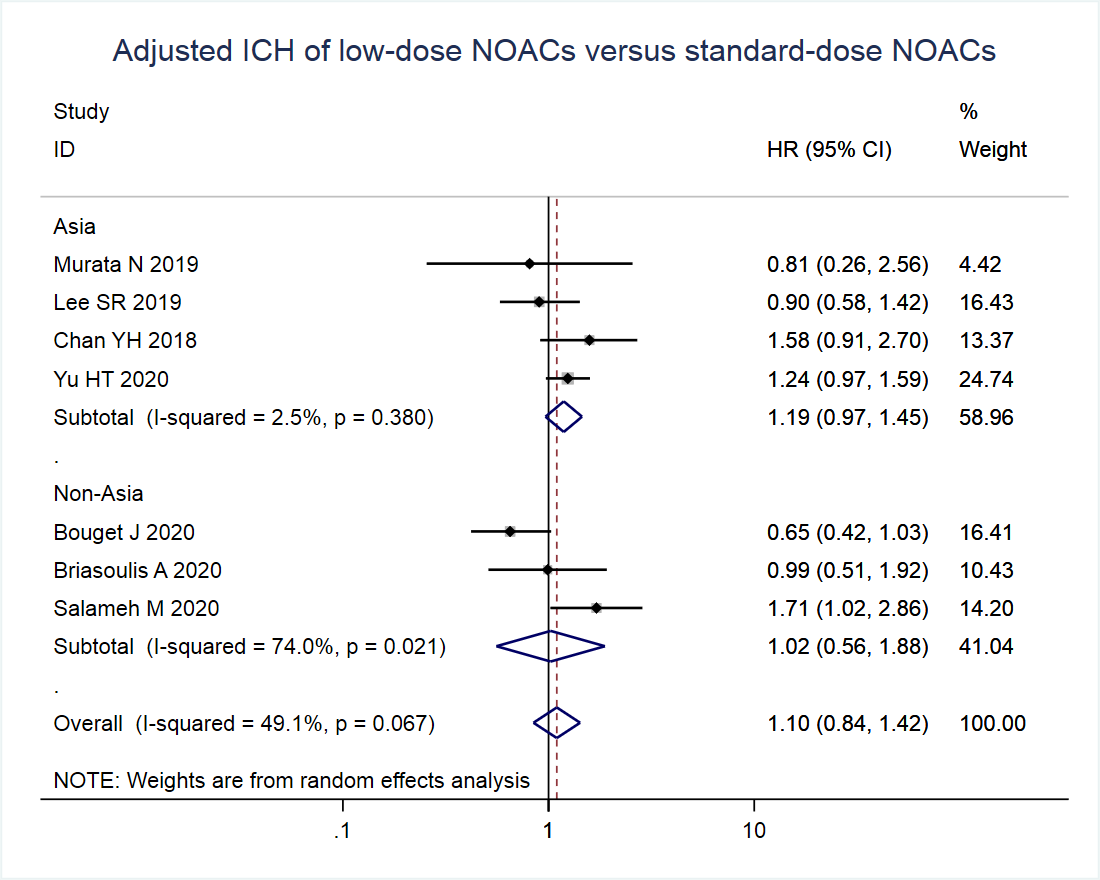


Figure S9. Pooled adjusted ICH of low-dose NOACs versus standard-dose NOACs

ICH = intracranial haemorrhage; HR = hazard ratio.


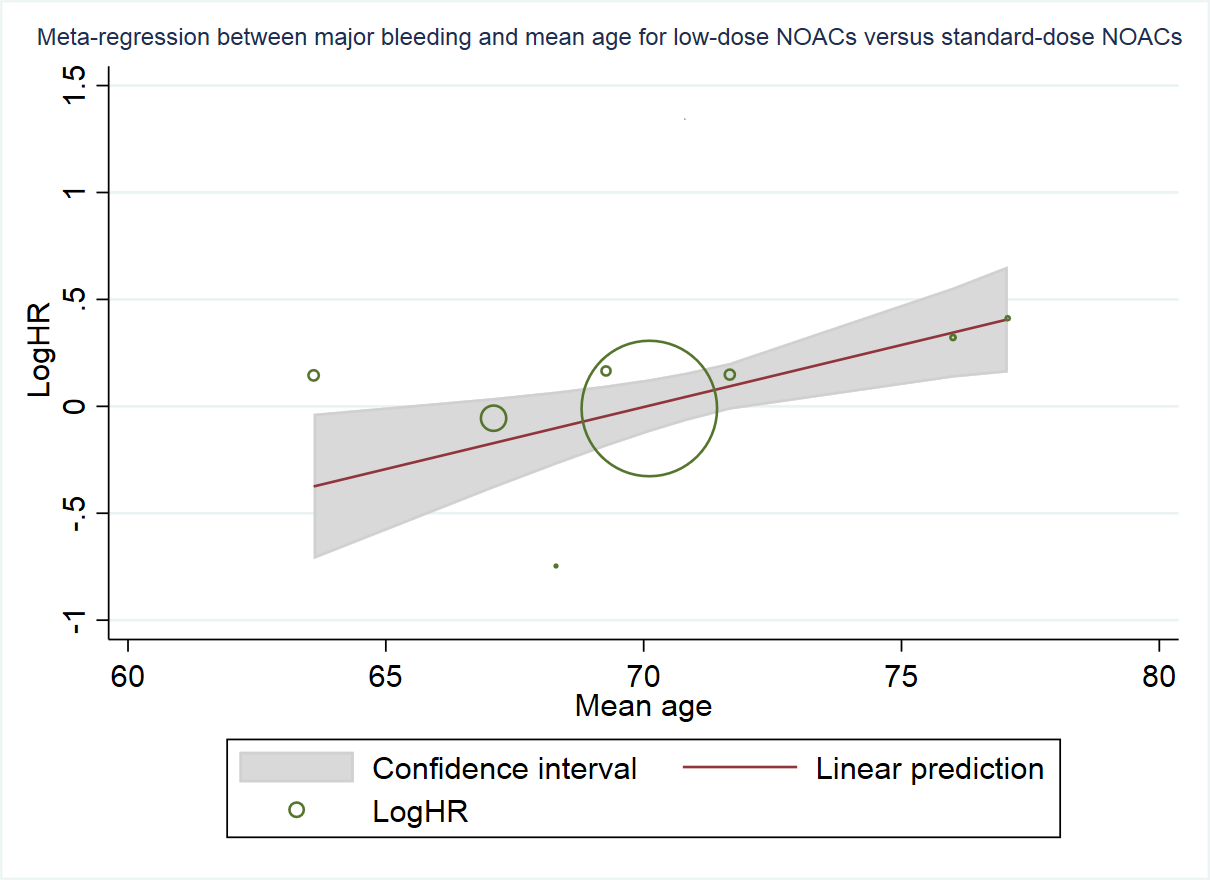


Figure S10. Result of meta-regression between major bleeding and mean age for low-dose NOACs versus standard-dose NOACs

HR = hazard ratio.


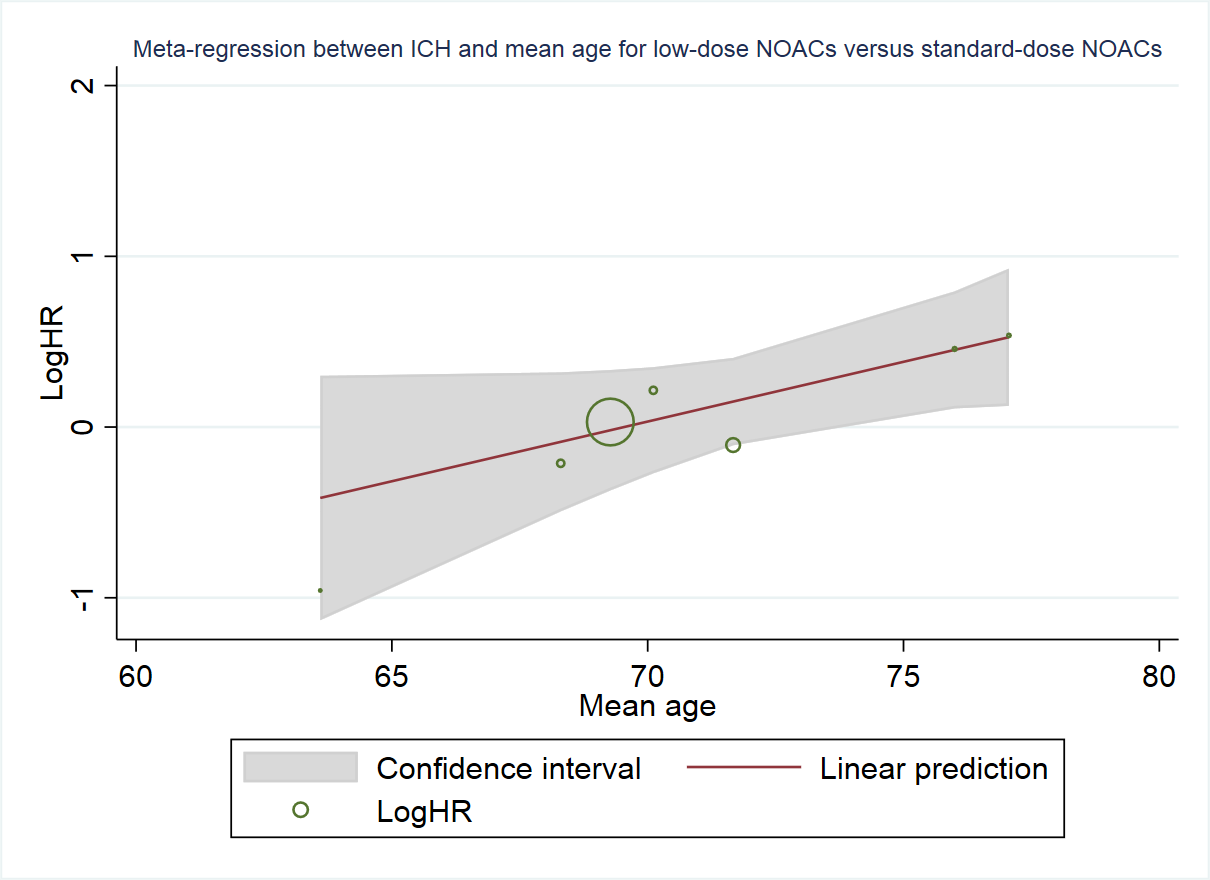
Figure S11. Result of meta-regression between ICH and mean age for low-dose NOACs versus standard-dose NOACs

HR = hazard ratio.


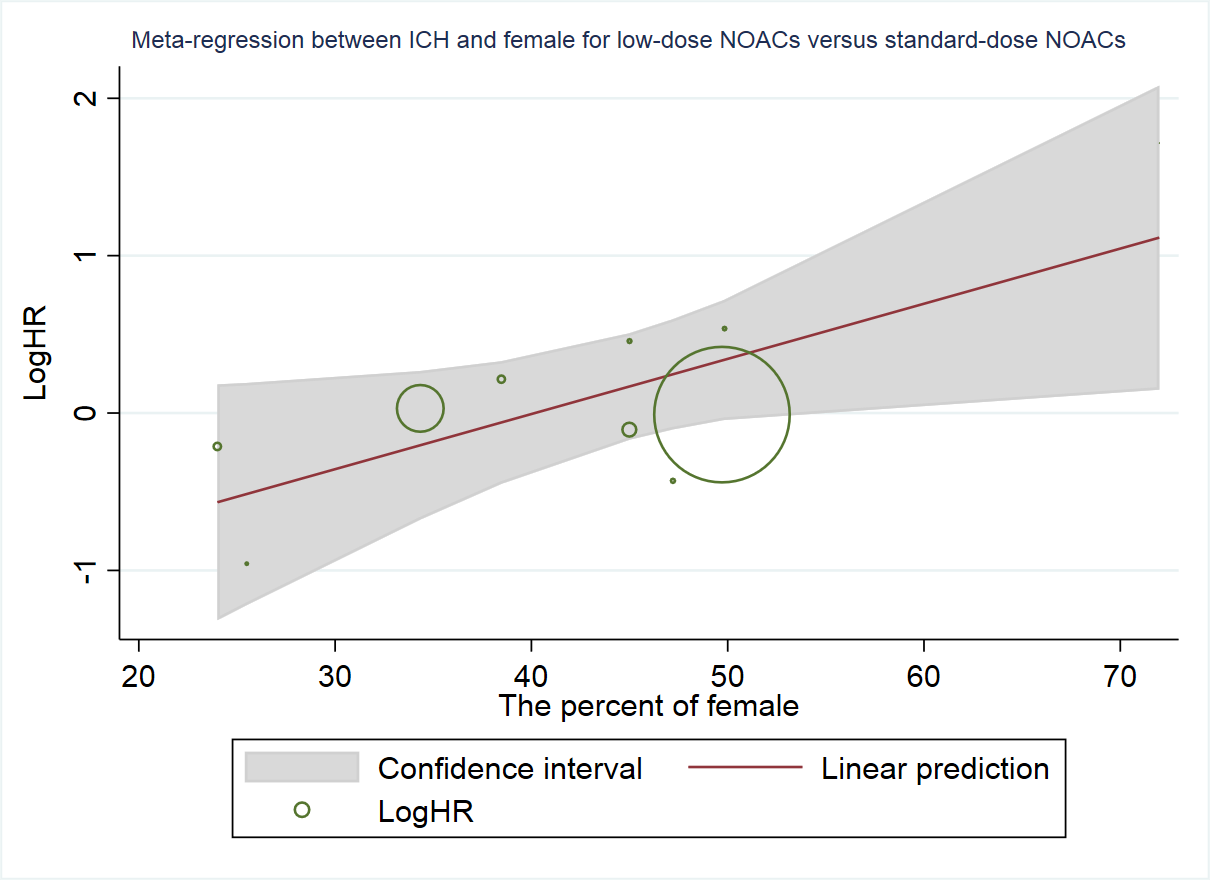
Figure S12. Result of meta-regression between ICH and female for low-dose NOACs versus standard-dose NOACs

HR = hazard ratio.


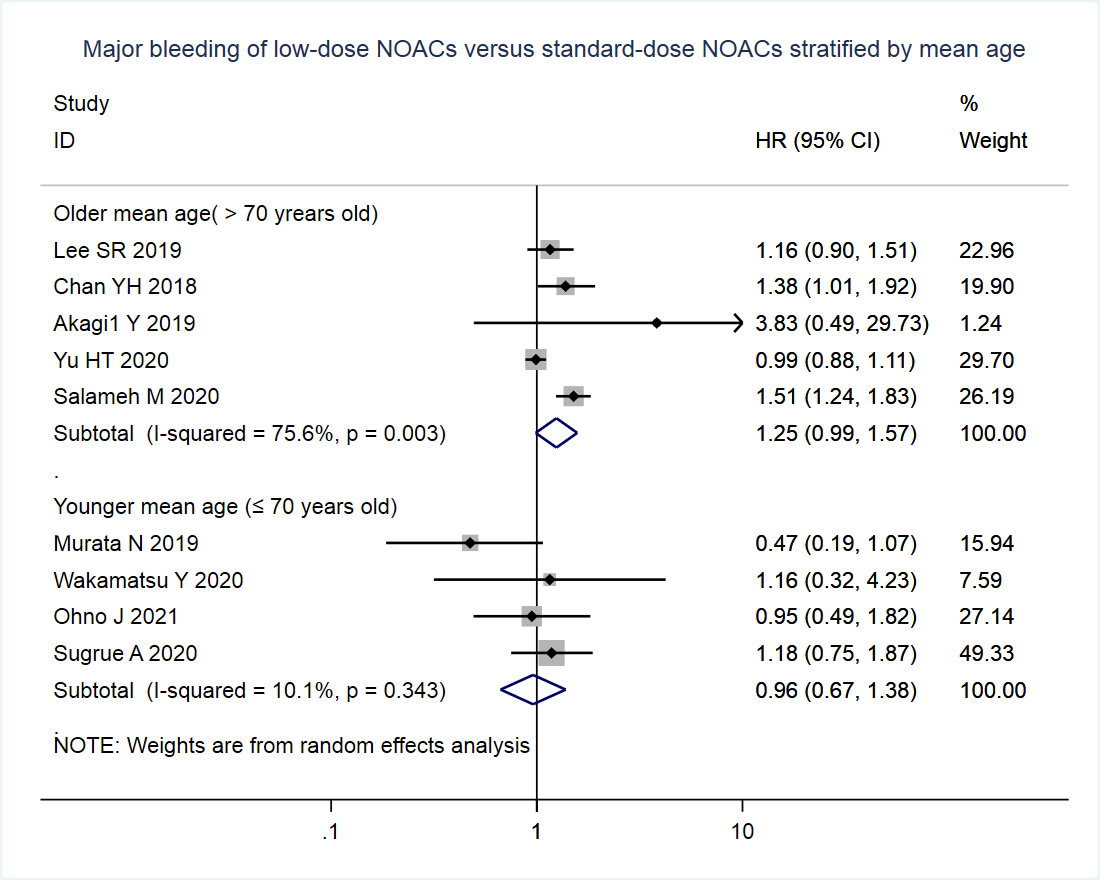


Figure S13. Pooled major bleeding of low-dose NOACs versus standard-dose NOACs stratified by mean age

HR = hazard ratio.


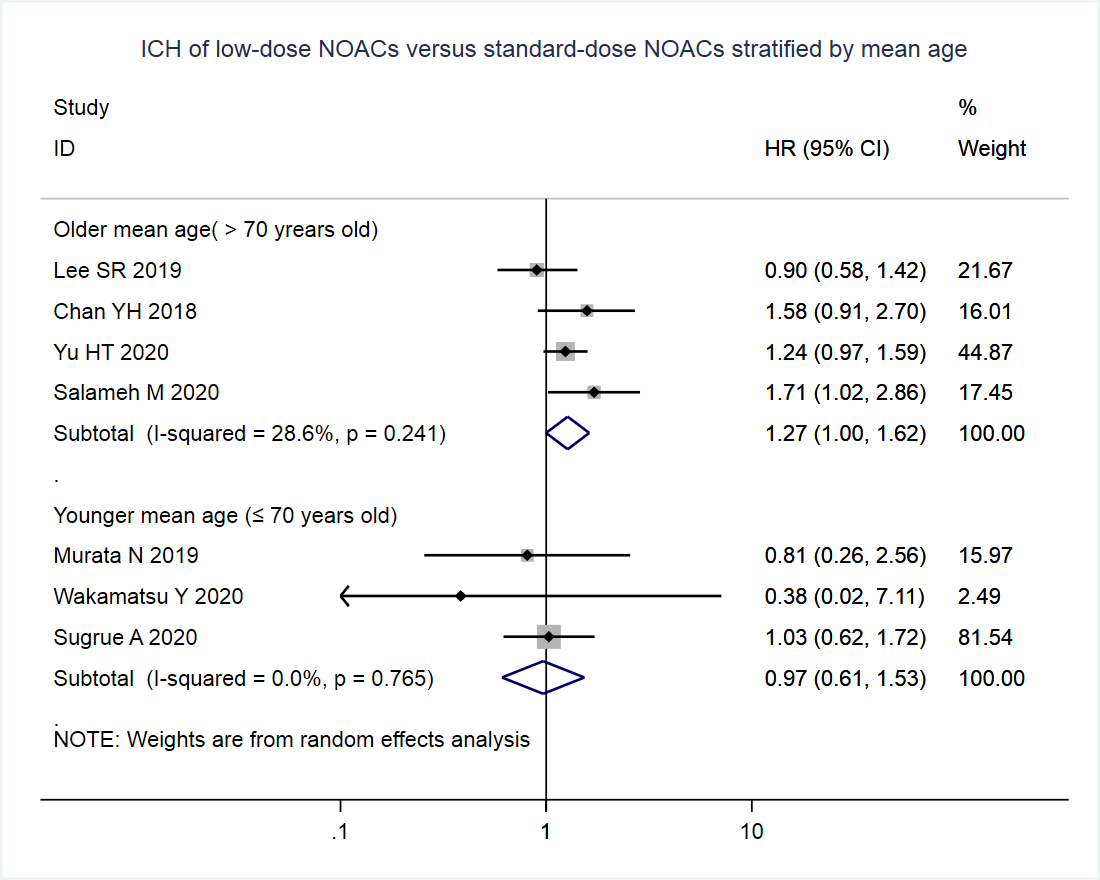
Figure S14. Pooled ICH of low-dose NOACs versus standard-dose NOACs stratified by mean age

HR = hazard ratio.


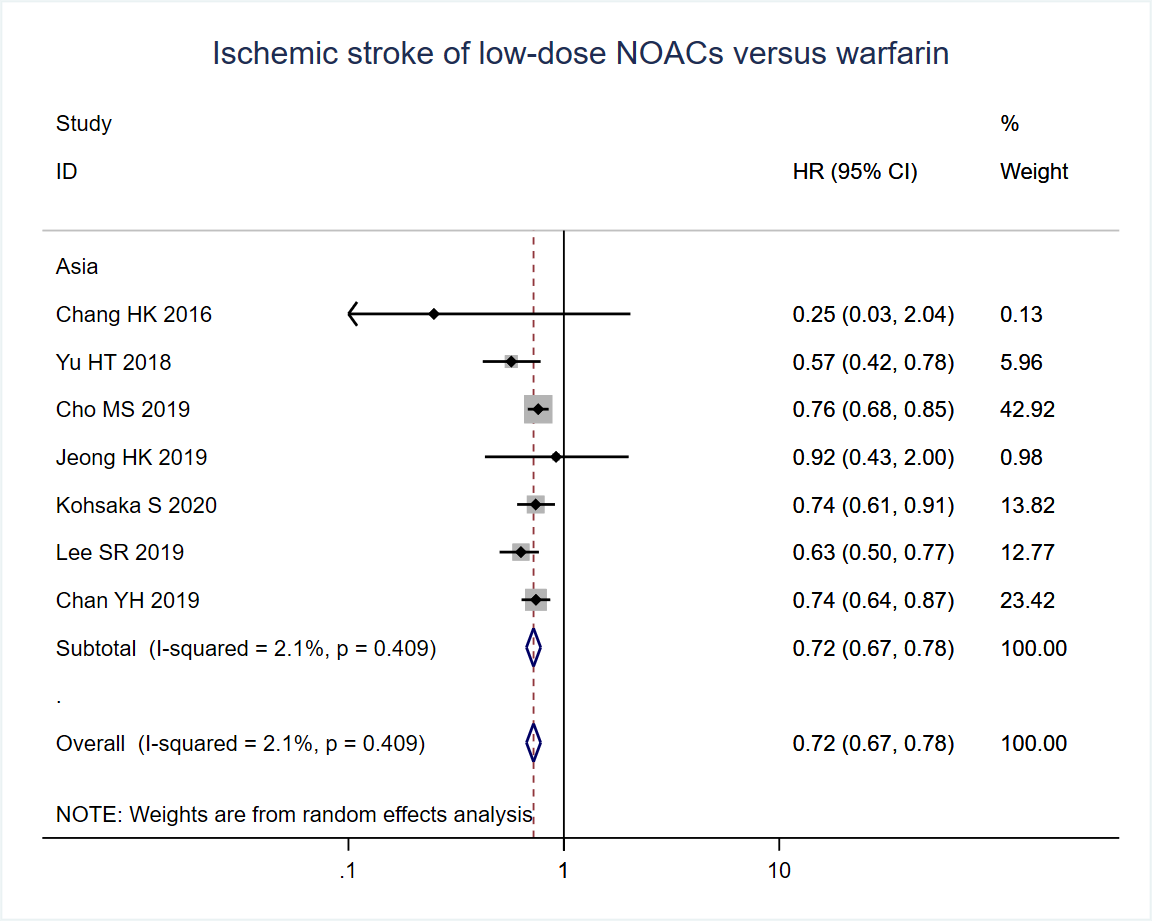


Figure S15. Pooled ischemic stroke of low-dose NOACs versus warfarin

HR = hazard ratio.


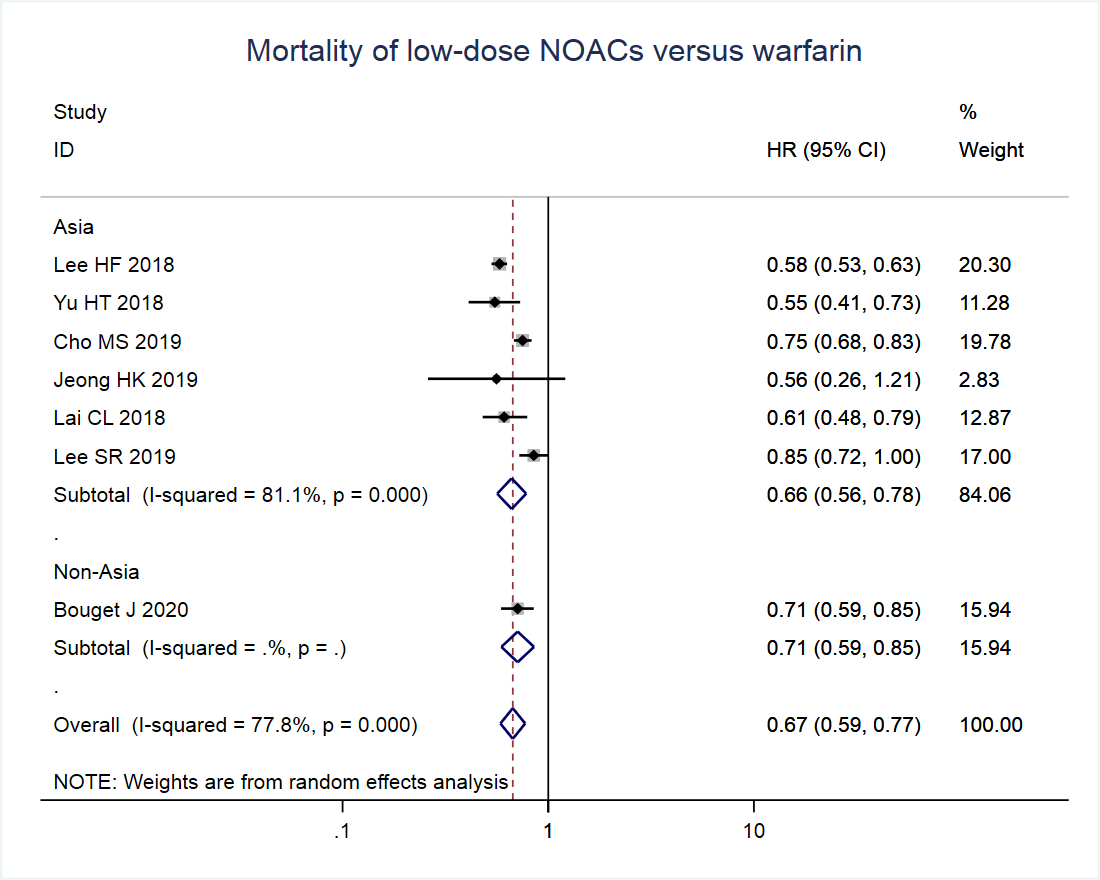
Figure S16. Pooled mortality of low-dose NOACs versus warfarin

HR = hazard ratio.


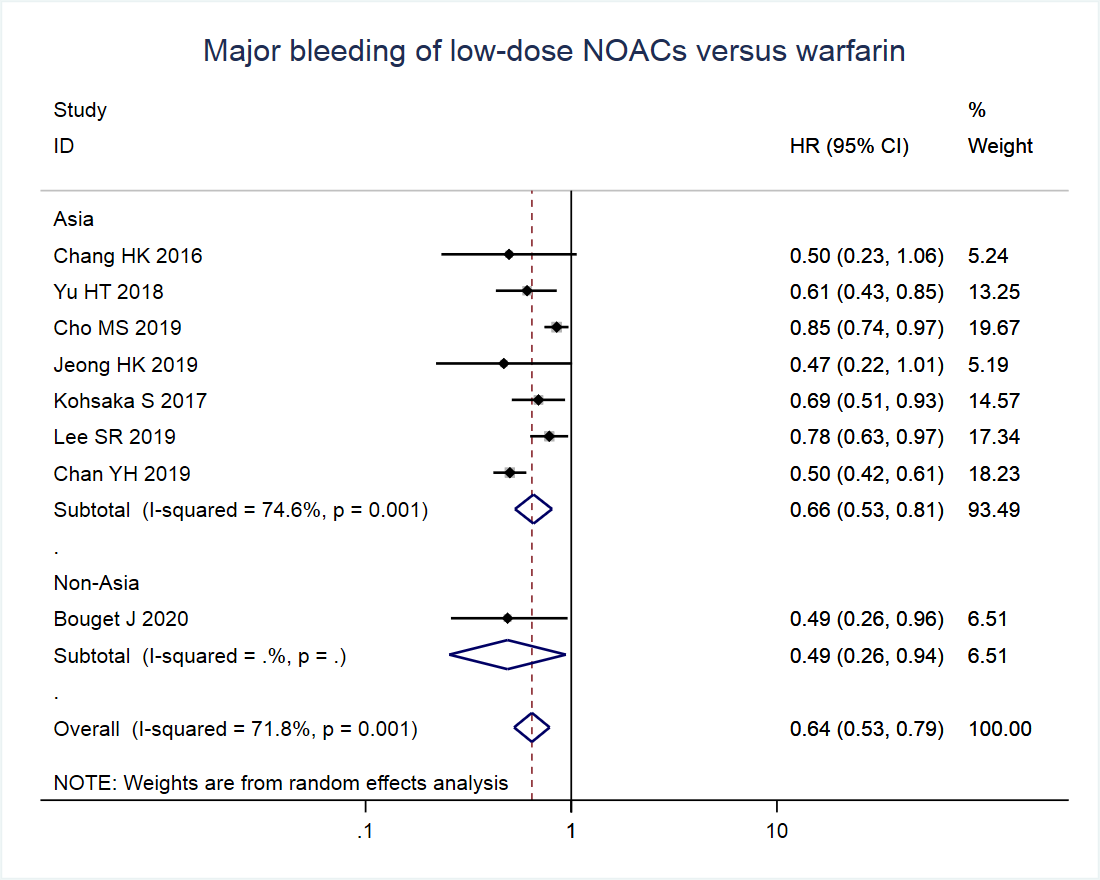
Figure S17. Pooled major bleeding of low-dose NOACs versus warfarin

HR = hazard ratio.


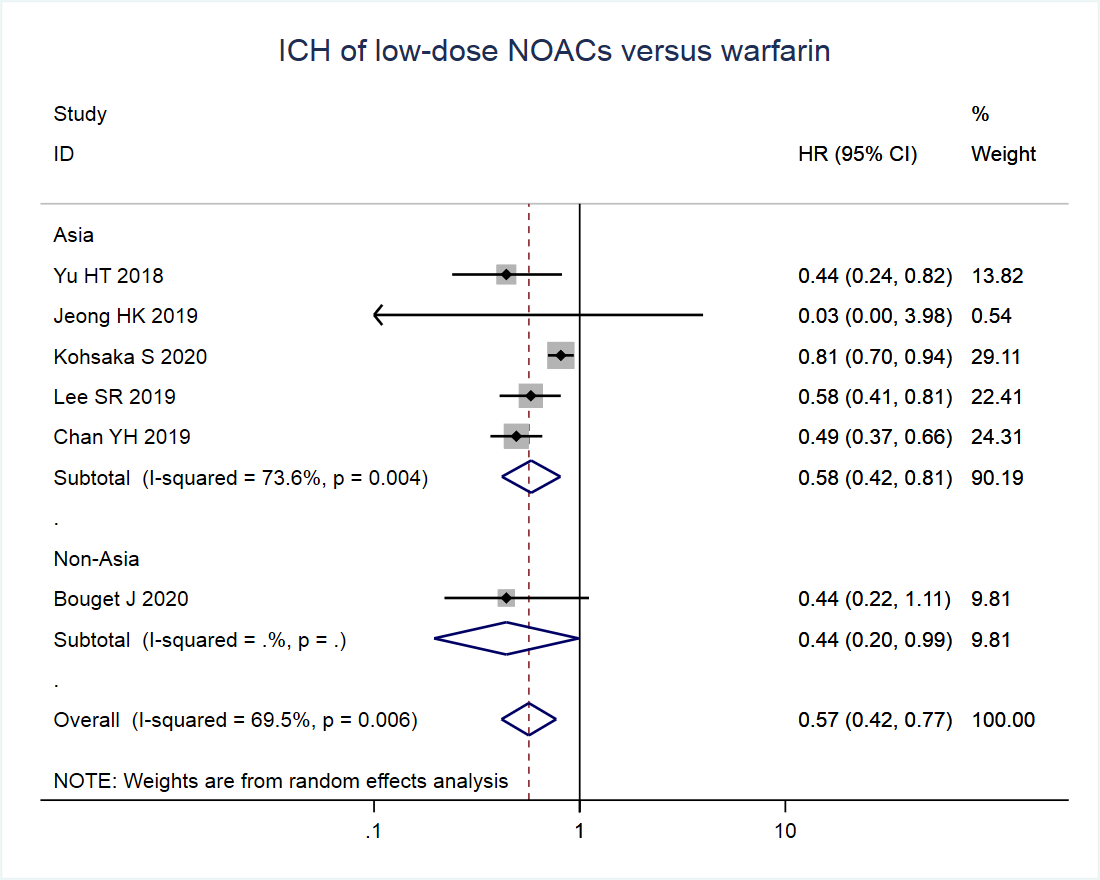
Figure S18. Pooled ICH of low-dose NOACs versus warfarin

ICH = intracranial haemorrhage; HR = hazard ratio.


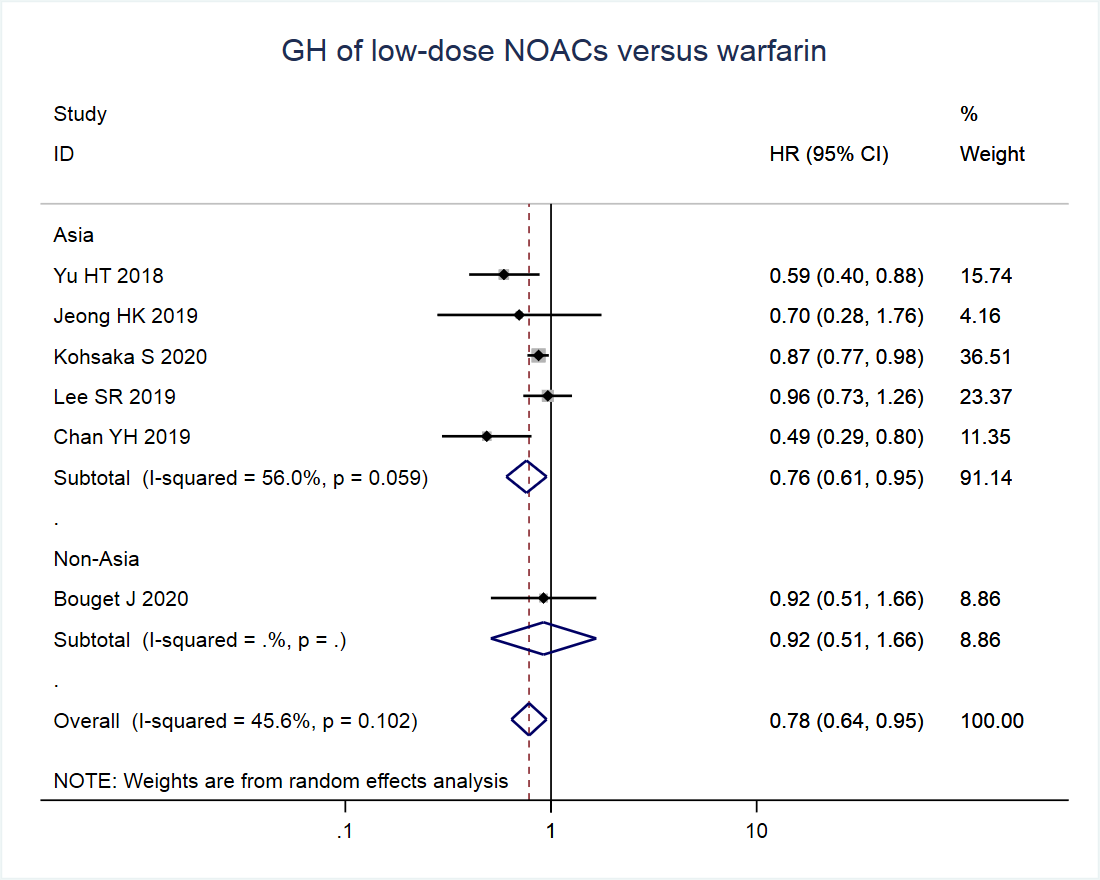
Figure S19. Pooled GH of low-dose NOACs versus warfarin

GH = gastrointestinal haemorrhage; HR = hazard ratio.


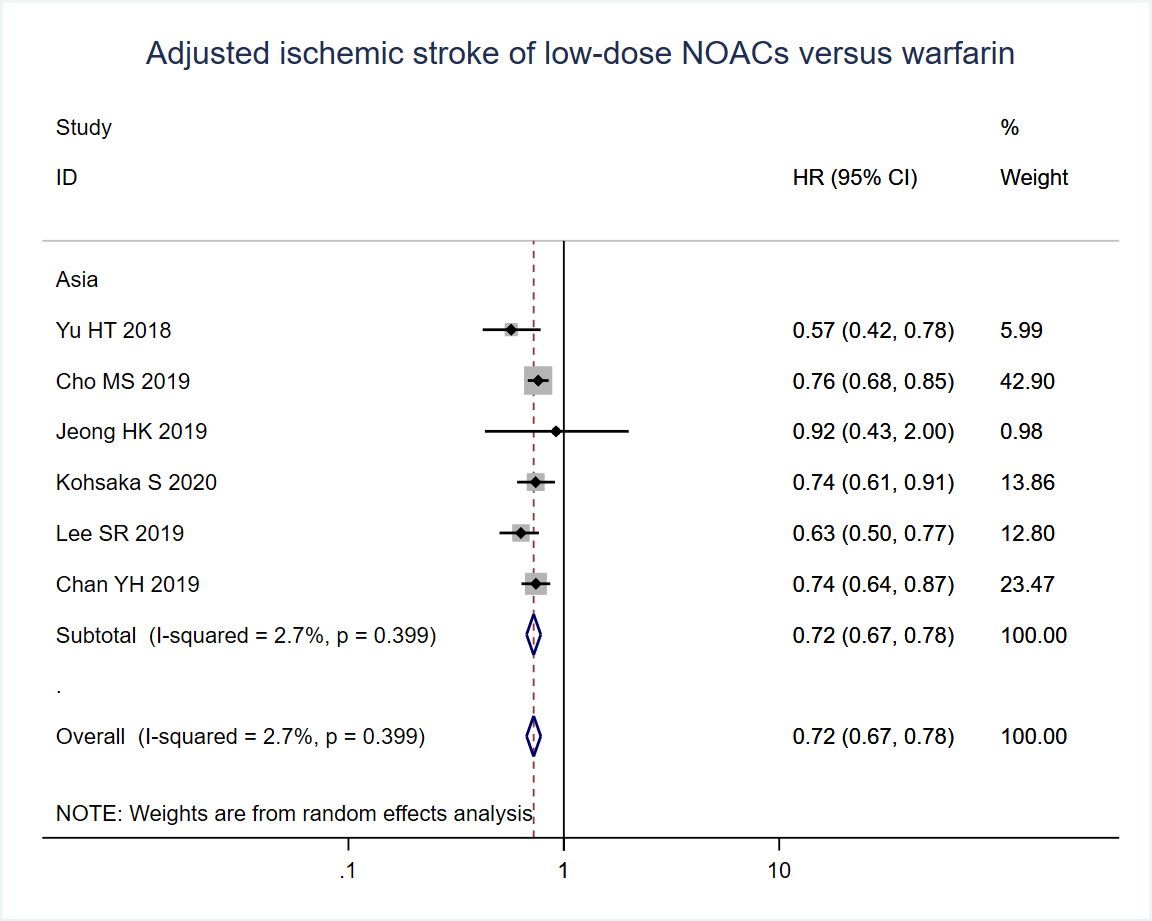


Figure S20. Pooled adjusted ischemic stroke of low-dose NOACs versus warfarin

HR = hazard ratio.


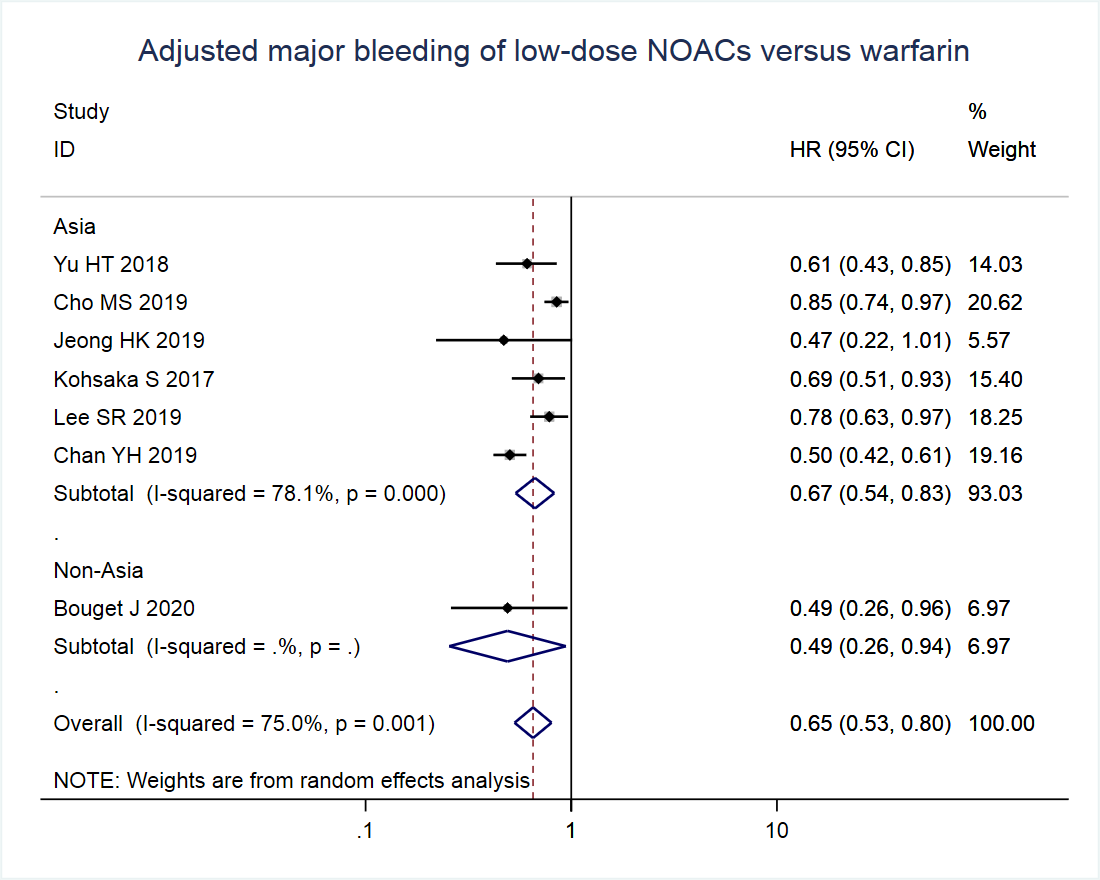
Figure S21. Pooled adjusted major bleeding of low-dose NOACs versus warfarin

HR = hazard ratio.
